# Photoreceptor Degeneration in Pro23His Transgenic Rats (Line 3) Involves Autophagic and Necroptotic Mechanisms

**DOI:** 10.3389/fnins.2020.581579

**Published:** 2020-11-03

**Authors:** Kiana Kakavand, Andrew I. Jobling, Ursula Greferath, Kirstan A. Vessey, Robb U. de Iongh, Erica L. Fletcher

**Affiliations:** ^1^Visual Neuroscience Laboratory, Department of Anatomy and Neuroscience, The University of Melbourne, Parkville, VIC, Australia; ^2^Ocular Development Laboratory, Department Anatomy and Neuroscience, The University of Melbourne, Parkville, VIC, Australia

**Keywords:** apoptosis, autophagy, cell death, necroptosis, retinitis pigmentosa photoreceptor

## Abstract

Photoreceptor death contributes to 50% of irreversible vision loss in the western world. Pro23His (P23H) transgenic albino rat strains are widely used models for the most common rhodopsin gene mutation associated with the autosomal dominant form of retinitis pigmentosa. However, the mechanism(s) by which photoreceptor death occurs are not well understood and were the principal aim of this study. We first used electroretinogram recording and optical coherence tomography to confirm the time course of functional and structural loss. Electroretinogram analyses revealed significantly decreased rod photoreceptor (a-wave), bipolar cell (b-wave) and amacrine cell responses (oscillatory potentials) from P30 onward. The cone-mediated b-wave was also decreased from P30. TUNEL analysis showed extensive cell death at P18, with continued labeling detected until P30. Focused gene expression arrays indicated activation of, apoptosis, autophagy and necroptosis in whole retina from P14-18. However, analysis of mitochondrial permeability changes (ΔΨm) using JC-1 dye, combined with immunofluorescence markers for caspase-dependent (cleaved caspase-3) and caspase-independent (AIF) cell death pathways, indicated mitochondrial-mediated cell death was not a major contributor to photoreceptor death. By contrast, reverse-phase protein array data combined with RIPK3 and phospho-MLKL immunofluorescence indicated widespread necroptosis as the predominant mechanism of photoreceptor death. These findings highlight the complexity of mechanisms involved in photoreceptor death in the Pro23His rat model of degeneration and suggest therapies that target necroptosis should be considered for their potential to reduce photoreceptor death.

## Introduction

Photoreceptor death causes approximately 50% of all cases of irreversible vision loss in the western world (Taylor et al., 2005). Retinitis pigmentosa (RP) is the most common form of inherited retinopathy, characterized by progressive loss of photoreceptors (rods and cones), which leads to severe visual impairment (Guadagni et al., 2015; Bravo-Gil et al., 2017). While approximately 96 different mutated genes have been identified in patients with RP (RetNet; November 2019)^1^, mutations in rhodopsin (*RHO*) are the most common causes of RP, accounting for approximately 15% of all inherited human retinal degenerations (Chapple et al., 2001) and 30–40% of patients with the autosomal dominant form of the disease (Ferrari et al., 2011). More than 150 different mutations have been identified in *RHO*, but the most frequent is a single-base C-to-A transversion, resulting in substitution of proline for histidine in codon 23 (P23H), causing autosomal dominant RP (Dryja et al., 1990; Athanasiou et al., 2018). P23H-RHO is thought to cause improper folding at the N-terminal, the binding site of 11-*cis*-retinal, resulting in the formation of dysfunctional, oligomeric aggregates within the endoplasmic reticulum (ER) and Golgi (Chapple et al., 2001). Although not entirely clear, it is likely that the resultant recruitment of ER-resident chaperones and activation of ubiquitin/proteasome system (Chapple et al., 2001) becomes overwhelmed resulting in ER stress and activation of the unfolded protein response (UPR), which in turn leads to photoreceptor death (Chiang et al., 2012, 2015, 2016; Athanasiou et al., 2017). However, the precise mechanisms that lead to photoreceptor loss are not well understood.

Rat models carrying a transgene expressing the mouse rhodopsin gene (*Rho*) with a C-A substitution at codon 23 (P23H amino acid conversion) were first described in Steinberg et al. (1996). Three different lines have been developed and while many studies have focused predominantly on line 1 (Cuenca et al., 2004, 2014; Pennesi et al., 2008; Garcia-Ayuso et al., 2013; Orhan et al., 2015), various studies have shown that line 3 (P23H-3) shows an intermediate rate of degeneration compared to lines 1 (which is more rapid) and 2 (slow). While transgene expression begins at P5, morphological change is not detectable until after P30, with the ONL decreasing in thickness to ∼85% of wild-type by P30, 50% by P120-P200 and ∼40% by P260 (Lewin et al., 1998; Machida et al., 2000; Pennesi et al., 2008). This progressive decline is matched by similar declines in retinal function with the rod a-wave showing a decline of 74% between 4 and 29 weeks (Machida et al., 2000; LaVail et al., 2018), though the effects of the mutation on retinal function appear to be affected by pigmentation and also copy number of the transgene (Cuenca et al., 2014).

The mechanism(s) of photoreceptor death in the P23H-3 rat are not well understood and many pathways have been implicated including oxidative stress, inflammation, ER stress, caspase-dependent and/or–independent pathways of apoptosis, necroptosis and autophagy. Autophagy is an important pathway for turning over the organelles within a cell and is known to be a critical factor in photoreceptor homeostasis. Indeed, inhibition of autophagy is known to reduce photoreceptor loss in the P23H-3 mouse model of retinal degeneration (Yao et al., 2018). Necrosis, which is characterized by swelling of the cytoplasm and organelles, followed by cell membrane disruption and release of the cytosolic constituents into the surrounding extracellular space, is associated with inflammation. In some circumstances, the inflammatory cytokines release from cells mediates a form of “programmed” necrosis called necroptosis (Shan et al., 2018).

In this study, we describe in detail the early time course and cell death mechanisms that result in photoreceptor dysfunction and loss in P23H-3 rats aged from postnatal (P) days P18 to P90 (3 months). Twin-flash ERG showed evidence of rod and cone photoreceptor dysfunction from P30, whereas structural decreases in the ONL [optical coherence tomography (OCT) and histology] were not evident until P60, with changes more pronounced in central and mid-peripheral retina. The first evidence of cell death occurred at P18, with large numbers of TUNEL^+^ cells detected in the ONL. However, analysis of mitochondrial permeability, caspase cleavage and apoptosis-inducing factor (AIF) localization suggest that neither caspase-dependent, nor independent apoptotic pathways are major contributors to photoreceptor cell death in the P23H-3 rat model. Expression profiling of the retina combined with protein analyses suggest that multiple pathways including apoptosis, autophagy and necroptosis are modulated in the P23H-3 retina, but that necroptosis is the over-riding mechanism by which photoreceptors die, consistent with another recent study (Viringipurampeer et al., 2019).

## Materials and Methods

### Animals

All animal procedures were approved by the Animal Ethics Committees of the University of Melbourne (AEC# 1312958 and #1614030) and were carried out in accordance with the Association for Research in Vision and Ophthalmology (ARVO) statement for the Use of Animals in Ophthalmic and Vision Research. Initial breeding stock of P23H-3 transgenic rats (Steinberg et al., 1996) on the Sprague–Dawley (SD) background, were provided by A/Prof. Krisztina Valter-Kocsi (Australian National University). SD albino rats were obtained from Animal Resource Center (ARC) (Murdoch, WA, Australia). Homozygous male P23H-3 transgenic rats were mated with wild-type SD female rats to generate hemizygous P23H-3 rats for study. Both male and female rats were used. All rats were bred and housed at The University of Melbourne Medical Animal Facility (Parkville, VIC, Australia) on a 12-h light/dark cycle (luminance levels inside cages ranged from 13 to 58 Lux). Food and water were available *ad libitum*.

### Retinal Function Using Electroretinography

We used the electroretinogram to measure retinal function of wild-type and P23H-3 rats (*n* ≥ 10) at P18, P30, P60, and P90, as described previously (Jobling et al., 2013; Vessey et al., 2015; Von Eisenhart-Rothe et al., 2018). There are a number of different ways of using the electroretinogram to measure rod and cone mediated responses (Kremers and Tanimoto, 2018). We used a paired flash paradigm to assess rod and cone mediated responses; the first light stimulus elicits a mixed rod and cone mediated waveform, whereas the second light stimulus elicits a cone-only response. Briefly, dark-adapted (12 h) rats were anesthetized by intraperitoneal (IP) injection of ketamine (60 mg/kg, Provet, Melbourne, VIC, Australia) and xylazine (5 mg/kg, Provet) and 0.5% proparacaine hydrochloride (Alcaine; Alcon Laboratories, French’s Forest, NSW, Australia) eye drops. The pupil was dilated using 1% Atropine sulfate (Alcon Laboratories) and 2.5% phenylephrine hydrochloride (Bausch & Lomb, Chatswood, NSW, Australia). The reference electrode was placed in the mouth and the recording electrode (Ag/AgCl) on the cornea. Twin 2.1 log cd.s/m^2^ full-field flashes with 0.8-s inter-stimulus interval (ISI), from a Nikon SB900 camera flash (Nikon, Lidcombe, NSW, Australia), were used to evaluate the contribution of rod and cone photoreceptors in the ERG response (Lyubarsky and Pugh, 1996; Phipps et al., 2004). Responses were amplified (gain × 5,000; −3 dB at 1 Hz and 1 kHz) using a ML132 BioAmp amplifier (ADInstruments, Castle Hill, NSW, Australia) and digitized at 10 kHz over a 250-ms period (ML785 Powerlab/8sp amplifier; ADInstruments). The stimulus and recording of the ERG were coordinated by Scope software version 3.6.10 (ADInstruments). To isolate rod responses, the cone responses were subtracted from the initial mixed responses (Phipps et al., 2004; Weymouth and Vingrys, 2008; Jobling et al., 2013; Vessey et al., 2015). ERG component analysis was completed using previously published equations and techniques (Hood and Birch, 1996; Weymouth and Vingrys, 2008). The rod a-wave photoreceptor responses (rod a-wave) were isolated and analyzed using a modified PIII model, to derive the PIII amplitude (PIII Rmax in μV) and sensitivity (m^2^cd^–1^s^–3^). The rod and cone post-photoreceptoral function (b-wave) was isolated and then fitted using an inverted γ function to generate the PII. From the PII fit, the amplitude of the PII response (PII amplitude in μV) and the time to peak (ms) were derived. For oscillatory potentials (OPs), summed amplitudes of OP 2, 3, and 4 were used. OP1 and OP5 of the rod OPs were excluded as they are often small and cannot be distinguished from system noise. For each animal, three individual measurements were represented as an average.

### Measurement of Retinal Structure Using Optical Coherence Tomography

After completion of ERG recording, OCT and fundus images were captured on a Micron III rodent fundus camera using Micron III software (Phoenix Technology Group, Pleasanton, CA, United States) as described previously (Von Eisenhart-Rothe et al., 2018).

### RNA Extraction and Quantitative Real-Time PCR

Rats, anesthetized by ketamine and xylazine (as above), were euthanized by intracardial injection of pentobarbitone (Lethabarb, Virbac Pty., Ltd., Milperra, NSW, Australia). Eyes were enucleated, anterior segments removed, and posterior eyecups from P14 and P18, P23H-3 and SD rats (*n* = 9 per group) were dissected in ice-cold M199 culture medium (Sigma-Aldrich) to separate retinae from RPE/choroid/sclera. Retinae were snap frozen in liquid nitrogen and total RNA was extracted using the RNeasy^®^ Mini Kit (Qiagen, Chadstone, VIC, Australia) with on-column DNase-I digestion as per manufacturer’s instructions. RNA quality and concentration were measured using an Agilent 2200 Tape Station (Agilent Technologies, Germany) and only samples with RIN >8 were used.

The rat cell death pathway finder RT^2^ profiler PCR array (Cat#: PARN-212Z, Qiagen, Melbourne, VIC, Australia) was used to profile expression of 84 genes involved in programmed cell death pathways. Three pooled RNA samples (*n* = 3 retinae/sample) from P23H-3 and SD rats at each age (P14, P18) were assayed. Total RNA (400 ng) from each sample was reverse transcribed using the RT^2^ First Strand cDNA Kit (Qiagen, Melbourne, VIC, Australia) according to manufacturer’s instructions. Negative controls for genomic DNA contamination, lacking RT enzyme were included. The cDNA samples were mixed with RT^2^ SYBR Green Master mix (Qiagen, Melbourne, VIC, Australia) and amplified in 384-well plates containing four replicates of 96 assays (84 different genes of interest, five control genes, one gDNA control, three RT controls and three positive PCR controls), using the Applied Biosystems ViiA 7 Real-Time PCR System at 95°C for 15 min, followed by 95°C for 15 s and 60°C for 1 min (40 cycles).

The quantitative PCR data were analyzed by the ΔΔCt method, using a commercial Excel template (Qiagen) with normalization to the average of five housekeeping genes and fold-change calculated as 2^–ΔΔCt^. While the pathways represented on the PCR array were known, we used the Ingenuity Pathway Analysis software (Qiagen) to predict whether these pathways were activated or inhibited using the Molecule Activity Predictor function.

### Reverse-Phase Protein Arrays (RPPA)

Sprague–Dawley and P23H-3 retinae (*n* = 6/group), dissected in cold phosphate buffer saline (PBS) from eyes of P18 rats, were homogenized in 60 μl of CLB1 lysis buffer (Zeptosens, Bayer; 7M Urea, 2M thiourea, 4% CHAPS, 1% dithiothreitol, 4 mM spermidine, 2% Pharmalyte^®^ with protease inhibitors added), samples were centrifuged at 7500 × *g* for 10 min in a benchtop centrifuge and the supernatant was transferred to a fresh tube for analysis by reverse phase protein arrays (RPPA) at the ACRF Translational RPPA platform at Peter MacCallum Cancer Centre (Parkville, VIC, Australia). After quantification of protein concentration by Bradford assay (Bio-Rad), samples were diluted serially using a Caliper ALH3000 liquid handling robot (Perkin Elmer), and spotted onto ZeptoChips (Zeptosens, Bayer, Witerswil, Switzerland) in duplicate using a Nano-Plotter-NP2.1 non-contact arrayer (GeSim, Radeberg, Germany). After incubating with pre-validated primary antibodies (1:500; See Supplementary Table S2) for 20 h, chips were washed and incubated with Alexa Fluor^®^-647 conjugated anti-rabbit IgG (#Z-25308; 1:1000; Thermo Fisher Scientific, Scoresby, VIC, Australia) for 4 h. Zeptosens instrument and software (version 3.1) were used to scan and calculate the relative fluorescence intensities. All data were normalized to the background values obtained with secondary antibody only.

### Immunofluorescence and TUNEL

Fresh eyecups from P14, P18, P30, and P60 P23H-3 and SD rats were isolated and dissected, as described above, to remove the anterior segments, prior to being fixed in different fixatives specific for immunofluorescence and TUNEL assays (Supplementary Table S3). Tissues were either fixed at room temperature in 4% paraformaldehyde in 0.1M phosphate buffer (PB; pH 7.4) for 30 min for frozen sections or in Davidson’s fixative (8% paraformaldehyde, 31.5% ethanol, 2M acetic acid) for 4 h for paraffin embedding. In some cases, the eyecups were incubated for 30 min at RT in 10 nM MitoTracker Orange CMTMRos (M7510; Thermo Fisher Scientific) to label mitochondria, prior to paraformaldehyde fixation and freezing. For frozen sections, paraformaldehyde-fixed eyecups were cryoprotected in a series of graded concentrations of sucrose (10, 20, 30% in PB), embedded in Tissue-Tek O.C.T compound (Sakura Seiki, Tokyo, Japan) and frozen in isopentane cooled by liquid nitrogen as described previously (Von Eisenhart-Rothe et al., 2018). Mid-sagittal frozen sections (12 μm) were collected on poly-L-lysine coated glass slides (Polysine^®^ Adhesion slides, Thermo Fisher Scientific) and kept at −20°C. Paraffin embedded, Davidson-fixed tissues were sectioned at 5 μm and mounted on poly-L-lysine coated glass slides.

Optimal immunofluorescence staining protocols varied, depending upon the antibody used (see Supplementary Table S3 for details of fixation, antigen retrieval and antibody dilutions). After incubation of sections overnight at 4°C with primary antibodies in blocking solution (PB, supplemented with 3% normal goat serum, 1% w/v bovine serum albumin (BSA), 0.05% w/v sodium azide, 0.5% v/v Triton-X), reactivity was detected by incubation with the appropriate species Alexa Fluor 488/594-conjugated antibodies (1:500 Invitrogen) in phosphate buffer containing 1 μg/ml 4′,6-diamidino-2-phenylindole (DAPI) (Thermo Fisher Scientific) to label cell nuclei. Following washes in PB, sections were cover-slipped with fluorescence mounting medium (Dako) and imaged by confocal microscopy (Zeiss LSM510 Meta, Carl Zeiss, North Ryde, NSW, Australia).

To label dying cells, TUNEL assay (DeadEnd^TM^ Fluorometric TUNEL System; Promega Corp., Alexandria, NSW, Australia) was performed on fixed-frozen sections as per manufacturer’s instructions. Briefly, sections were washed in PBS, immersed in 0.2% Triton-X in PBS for 20 min and incubated in equilibration buffer (Promega, Alexandria, NSW, Australia) for 10 min prior to the rTdT reaction at 37°C for 1 h in the dark. Subsequently, sections were washed 15 min in 2× SSC and PBS (3 × 5 min) before a counter-stain with 1 μg/ml DAPI (Thermo Fisher Scientific) in PBS for 20 min. Sections were cover-slipped and imaged by confocal microscopy as described above.

To quantify photoreceptor death, TUNEL^+^ cells in the ONL were counted in three mid-sagittal eye sections per animal (*n* > 5/group). Each half retina (from optic nerve to periphery) was divided into three segments (central, mid-peripheral and peripheral regions) and using ImageJ software the number of TUNEL^+^ was expressed per millimeter of retinal length. Similarly, the mean thicknesses of the ONL, INL, and GCL were measured in each segment using Image J.

To label cone photoreceptors, frozen sections were incubated with FITC-conjugated peanut agglutinin (PNA) used at 1:500 (Vector Laboratories, Burlingame, CA, United States) in PBS. The number of PNA^+^ cells were quantified across the whole retina.

### Mitochondrial Permeability Assay

To analyze mitochondrial membrane potential changes (ΔΨm) in the P23H-3 retina, the MitoProbe^TM^ JC-1 Assay Kit (Thermo Fisher Scientific) was performed according to the manufacture’s instruction with minor modifications. Briefly, retinae from P23H-3 and SD rats at P14 and P18 (*n* = 6 per group) were isolated as above, cut into small pieces using a scalpel and dissociated into single cells using the Papain Dissociation System (Worthington Biochemical Corporation, Lakewood, NJ, United States). Cells obtained from each sample were counted on a Countess^TM^ automated cell counter (Thermo Fisher Scientific) and 1 × 10^6^ cells from each sample were suspended in 1 mL warm PBS, containing 0.5 μM JC-1 dye and incubated 30 min at 37°C with 5% CO_2_. A positive control sample was incubated with a mitochondrial depolarizing agent (0.5 mM CCCP) prior to JC-1 staining. After washing in warm PBS, cells were centrifuged at 300 × *g* for 5 min and resuspended in 200 μL PBS. Prior to analysis on a flow cytometer (BD FACS Aria III Cell Sorter), cells were stained with DAPI as an indicator of cell viability. Initial gating by forward-scatter (FSC) versus side-scatter (SSC) distinguished three distinct subpopulations: small dead cells, small live cells, and large live cells (Supplementary Figure S3), before analysis with 488 nm excitation and emission filters Alexa Fluor 488 dye (530 nm) and R-phycoerythrin (695 nm). Data were analyzed using FlowJo (V.10.4.2) software.

Based on previous studies, isolated photoreceptors are characteristically smaller than other retinal cells due to being terminally differentiated (Akimoto et al., 2006). To determine which populations of cells in our flow cytometry analysis were enriched for photoreceptors, we prepared single cell suspensions of retinal cells isolated from P16 P23H-3 rats (*n* = 2), stained with DAPI and separated as described above. RNA was extracted from isolated cell populations using the RNeasy Micro Kit (Qiagen, Hilden, Germany) and purity confirmed on an Agilent Tape Station as described above. cDNA library preparation and pre-amplification was performed using the Smart-seq2 protocol (Picelli et al., 2014) with 9 cycles of pre-amplification. Quantitative PCR analysis was performed using a Rotor gene 3000 PCR cycler (Qiagen, Hilden, Germany) using specific primers for rat *Rho* (Forward 3′-AGCAACAGGAGTCGGCTACC-5′; Reverse 3′-CCGAAGTTGGAGCCCTGGTG-5′), *Opn1mw*, (Fw 3′-GTCCAGACGTGTTCAGCG; Rev 3′-GACCATCACCACCACCAT-5′) *Opn1sw* (Fw 3′-CAGCCTTCATGGGATTTG; Rev 3′-GTGCGTGCTTGGAGTTGA) and *Hprt* (Fw 3′-CCTAAAAGACAGCGGCAAGTTGAA; Rev 3′-CCACAGGACTAGAACGTCTGCTAG). Relative expression was calculated using the 2^–ΔΔ*Ct*^ method.

### Statistical Analysis

Analysis and curve fitting of the ERG data was carried out in Excel (Microsoft, Redmond, WA, United States). Statistical analysis and graphing were performed using Graph Pad Prism 7 (San Diego, CA, United States). All data are expressed as mean ± standard error of the mean (SEM). Two-way analyses of variance (ANOVA) were used to determine statistical difference among experimental groups where the two independent variables were genotype and age, followed by Tukey’s *post hoc* tests for further pair-wise comparisons. Comparisons of fluorescence ratios from P23H-3 and SD flow-sorted cells were conducted using two-tailed, unpaired *t*-tests. *p*-values less than 0.05 were considered statistically significant and represented as ^∗^*p* < 0.05, ^∗∗^*p* < 0.01, ^∗∗∗^*p* < 0.001, and ^****^p < 0.0001 in all graphs.

## Results

### Structural and Functional Changes in the Retina Occur in the P23H-3 Rat From an Early Age

**FIGURE 1:**
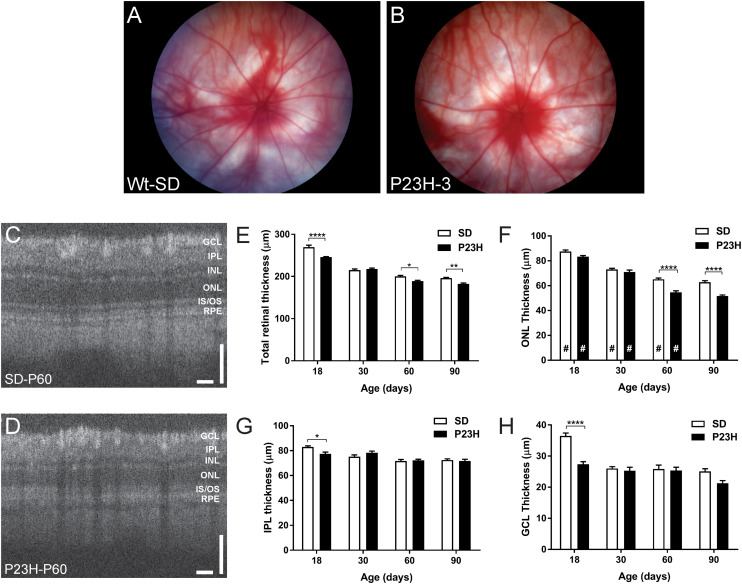
Fundus and OCT imaging images of P23H-3 and SD rat retinae. Representative fundus images SD **(A)** and P23H-3 **(B)** rats at P60. No morphological differences were apparent in fundus scans of P23H-3 rats at any age, compared to age-matched SD rats. Representative OCT images of the central retina in SD **(C)** and P23H-3 **(D)** rats at P60. Segmentation analyses of OCT images showing mean thicknesses of total retina **(E)**, ONL **(F)**, IPL **(G)**, and GCL **(H)**. Total thicknesses of P23H-3 retinae were significantly thinner than SD at P18, P60, and P90 **(E)**. At P18 this was attributable to changes in the thickness of IPL **(G)** and GCL **(H)** but not the ONL **(F)**. Changes in the ONL were only detected at P60 and P90 **(F)**. All histograms indicate mean ± SEM, two-way ANOVA, Tukey’s *post hoc* test, *p < 0.05, **p < 0.001, and ****p < 0.0001, *n* ≥ 8 in each group. *: significant difference for genotype, #: significant difference for age. Abbreviations: ONL, outer nuclear layer; INL, inner nuclear layer; IPL, inner plexiform layer; GCL, ganglion cell layer. Scale bar **(C,D)** = 100 μm.

The central aim of this study was to examine the mechanisms by which photoreceptor death develops in the P23H-3 model of retinal degeneration. We first evaluated retinal function and structure using electroretinogram (ERG) recording and OCT. Fundus imaging showed no overt alterations in the retina or presence of distinct pathological features at P18, P30, P60 (Figures 1A,B) or P90. By contrast, OCT imaging (Figures 1C,D) clearly revealed reductions in total retinal thickness at P18, P60, and P90 (Figure 1E). Quantification of total retinal thickness showed a reduction at P18 (Figure 1E) that was attributed to significant thinning of the IPL and GCL (Figures 1G,H) but not the ONL (Figure 1F). In contrast, a redution in total retinal thickness at P60 and P90 was associated with a significant decreases in ONL thickness at these ages (Figure 1F).

**FIGURE 2:**
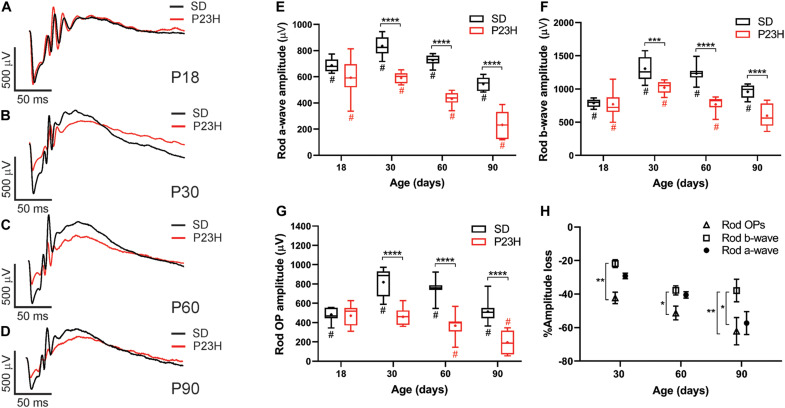
Alterations in rod responses of P23H-3 rats with age. **(A–D)** Representative rod responses in SD and P23H-3 rats at P18 **(A)**, P30 **(B)**, P60 **(C)**, and P90 **(D)**. **(E–G)** Box and whisker plots of rod ERG responses showing change in rod a-wave amplitudes **(E)**, rod b-wave amplitudes **(F)**, and rod OP amplitudes **(G)**. Rod a-wave amplitudes were significantly reduced in the P23H-3 compared to the SD from P30 **(E)**. The rod b-wave amplitudes were significantly attenuated in the P23H-3 rats from P30 onward **(F)**. The rod a-wave (PIII) **(E)**, rod b-wave (PII) **(F)**, and summed rod OP **(G)** showed a progressive age-related reduction from P30 to P90 in both strains. All box and whisker plots show interquartile range (box), median (transverse line), mean (+) and 95% confidence intervals (error bars). **(H)** Relative percentage changes in rod a-wave, b-wave, and summed OP responses. At P30 and P60, the a-wave and b-wave responses were reduced in P23H-3 to a similar extent. However, the loss of summed OP amplitudes was significantly increased compared to those of b-wave. At P90, the extent of the b-wave loss was significantly less than a-wave and summed OP. Data were analyzed by two-way ANOVA with Tukey’s *post hoc* test. *p < 0.05, **p < 0.01, ***p < 0.001, and ****p < 0.0001, *n* ≥ 8 in each group. *, significant difference for genotype; #, significant difference for age.

Twin flash ERG was used to assess rod- and cone-mediated function in P23H-3 and SD rats from P18. Representative rod-mediated waveforms for the SD and P23H-3 rats are shown in Figures 2A–D. No apparent difference in response was observed at P18, whereas at P30, P60, and P90, the amplitudes of the responses in P23H-3 rats were reduced compared to age-matched SD rats. Quantification of rod a-wave amplitudes showed a developmental increase in the rod responses from P18 to P30 in SD control which was not evident in P23H-3 rats (Figure 2E). The developmental increase in photoreceptor response has been reported to coincide with the post-natal elongation of the outer segments after eye opening (Fulton et al., 1995). While both strains showed a progressive a-wave decrease with age (*p* < 0.0001), the photoreceptor responses in the P23H-3 retina were significantly reduced (*p* < 0.0001) compared to SD responses from P30 onward (Figure 2E).

The rod b-wave responses, representing rod bipolar cell activity, were significantly reduced (*p* < 0.0001) in P23H-3 compared to SD from P30 onward and both P23H-3 and SD b-waves exhibited an age-related decline in amplitude from P30 (Figure 2F). A similar pattern was observed in the summed OP amplitude (Figure 2G), a waveform that is thought to reflect amacrine cell function. Unlike the rod photoreceptor amplitudes, rod implicit times remained unaffected at all ages (Supplementary Figure S1B) while rod sensitivity was only significantly different at P18 (Supplementary Figure S1A). As the ERG response is serial in nature, it is possible that the inner retinal changes (b-wave and OP) reflect altered photoreceptoral response. To explore this, we compared the percentage changes in rod (a-wave) with change in rod post-photoreceptor (b-wave, and summed OP) responses (Figure 2H). Overall, the changes in rod a-wave and rod b-wave at P30 and P60 were similar, suggesting that loss of photoreceptor function is the predominant functional change occurring within the retina. However, at these ages the OP responses were reduced to a greater extent, suggesting that in the P23H-3 rat there is photoreceptoral loss, as well as inner retinal changes affecting amacrine cell function. Together, these data show that retinal dysfunction occurs from P30 in the P23H-3 rat, with a specific photoreceptor and amacrine deficit evident.

**FIGURE 3:**
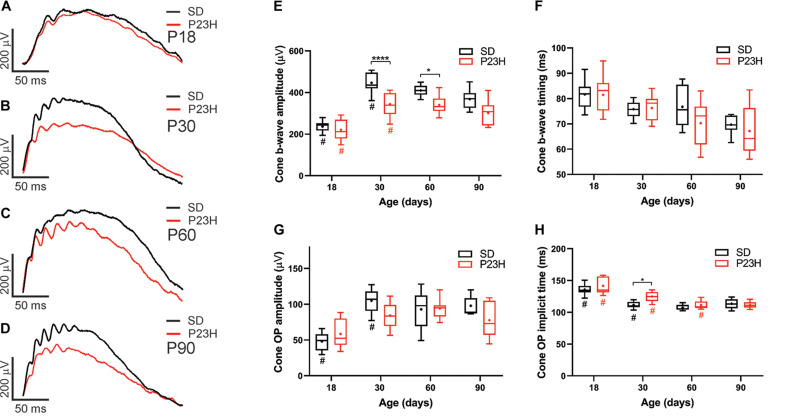
Alterations in cone responses of P23H-3 rats with age. Representative modeled cone responses in SD and P23H-3 rats at P18 **(A)**, P30 **(B)**, P60 **(C)**, and P90 **(D)**. **(E–H)** Box and whisker plots of cone ERG responses in SD and P23H-3 rats showing change in cone b-wave amplitudes **(E)**, cone b-wave time-to peak **(F)**, cone OP amplitude amplitudes **(G)**, and cone OP implicit times **(H)**. Cone b-wave amplitudes were significantly lower in P23H-3 compared to age-matched SD controls at P30 and P60 **(E)**. The cone b-wave timing and summed OP amplitudes were not significantly affected in P23H-3 at any age examined **(F,G)**. OP implicit timing showed a significant increase in P23H-3 compared to SD only at P30 **(H)**. All box and whisker plots show interquartile range (box), median (transverse line), mean (+), and 95% confidence intervals (error bars). Data were analyzed by two-way ANOVA, Tukey’s *post hoc* test, *p < 0.05, and ****p < 0.0001, *n* ≥ 8 in each group. *, significant difference for genotype; #, significant difference for age.

While cone photoreceptor function cannot be measured directly in rats, the cone-mediated post-photoreceptoral response (cone b-wave) indicated smaller cone ERG responses in P23H-3 from P30 compared to age-matched controls (Figures 3A–D). Quantification showed a developmental increase in cone responses from P18 to P30 in both SD and P23H-3 rats (Figure 3E). However, there were significant reductions in P23H-3 cone responses when compared to SD retinae at P30 and P60, but not at P90 (Figure 3E). The lack of difference at P90 may reflect the greater age-related decline in the SD cone response compared to P23H-3 and the difficulty in measuring the cone b-wave amplitudes. In contrast to the cone b-wave amplitudes, the cone b-wave implicit time (Figure 3F), OP amplitudes and timing remained unaffected in the P23H-3 retinae compared to the SD rats (Figures 3G,H). While these data show that cone photoreceptor responses are altered from P30 onward, counts of PNA^+^ cells at P30 showed no significant difference (Supplementary Figure S2G), suggesting changes are functional and not due to cell death.

### Photoreceptor Degeneration Is Evident From P18 in P23H-3 Rats

**FIGURE 4:**
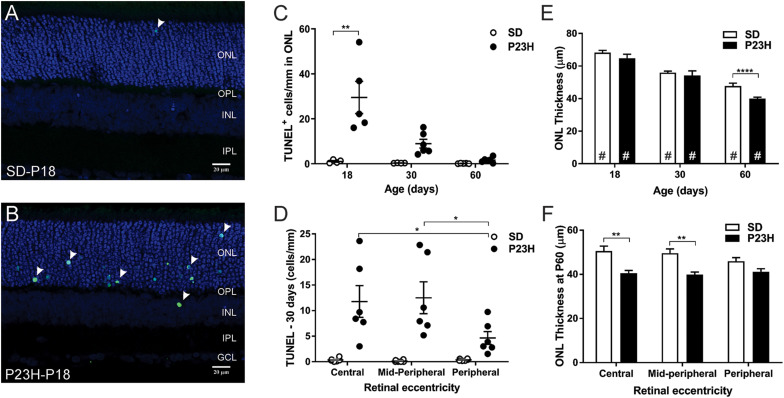
Photoreceptor cell death in P23H-3 rats with age. TUNEL (green) and nuclei (blue) staining in SD **(A)** and P23H-3 **(B)** retina at P18 **(A,B)**. TUNEL positive cells (arrowheads) were detected predominantly in ONL of P23H-3 retinae across all ages but were rarely detected in the ONL of SD retinae. **(C)** Quantification of total retinal TUNEL^+^ cells at each age revealed significantly increased numbers of TUNEL^+^ cells in P23H-3 retina at P18, but not at P30 or P60. **(D)** Significant differences in the number of TUNEL^+^ cells across retinal eccentricity were detected in the P23H-3 retina at P30 with greater photoreceptor death in central and mid-peripheral than in peripheral regions of retina. **(E)** Quantification of mean ONL thickness showed significant decreases with age (#), with P23H-3 ONL significantly thinner than SD at P60, but not at P18 or P30. **(F)** Across central to peripheral eccentricity at P60 there were significant decreases in ONL in the central and mid-peripheral, but not peripheral regions. Data are expressed as means ± SEM, Two-way ANOVA, Tukey’s *post hoc* test, *p < 0.05, **p < 0.001, and ****p < 0.0001, *n* ≥ 5 in each group. ^∗^: significant difference for genotype, ^#^: significant difference for age. Abbreviations: ONL, outer nuclear layer; INL, inner nuclear layer. Scale bar = 20 μm.

TUNEL assays were used to identify the time-course of photoreceptor degeneration in P23H-3 rat retina. In contrast to SD retinae, abundant labeled cells were observed in the ONL of the P23H-3 retinae at P18 (Figures 4A,B). Quantification of TUNEL^+^ cells showed that at P18 an average of ∼30 cells/mm were detected in the P23H-3 retina, which declined with age to approximately 8 cells/mm of retina at P30 and 1–2 cells/mm at P60 (Figure 4C). Similar to the OCT analyses (Figures 1C–H), measurement of ONL thickness in these sections showed progressive age-related decreases in ONL thickness from P18 to P60 in both SD and P23H-3 (Figure 4E) and at P60 the ONL was significantly thinner in P23H-3 than in SD rats. Further analyses across retinal eccentricity showed no significant differences in numbers of TUNEL^+^ cells in control SD retinae across eccentricity at any age (Figure 4D and Supplementary Figures S2A,B). By contrast in P23H-3 rats, while there were no differences in TUNEL^+^ cells at P18 or P30 across eccentricity (Supplementary Figures S2A,B), at P60 mean number of TUNEL^+^ photoreceptor cells in the peripheral retina was significantly lower than in the central and mid-peripheral regions (Figure 4D). Consistent with this, there were significant reductions in ONL thickness in central and mid-peripheral, regions of the retina at P60, but not at P18 or P30 (Figure 4F). No significant differences in ONL thickness were detected in P18 or P30 retinae at any eccentricity (Supplementary Figures S2C,D). While there were significant age-related decreases in INL and GCL thickness with age, no differences were observed in INL or GCL thickness between WT and P23H3 retinae at P18, P30 or P60 (Supplementary Figures S2E,F).

### Identification of Cell Death Pathways in the P23H-3 Rat

**FIGURE 5:**
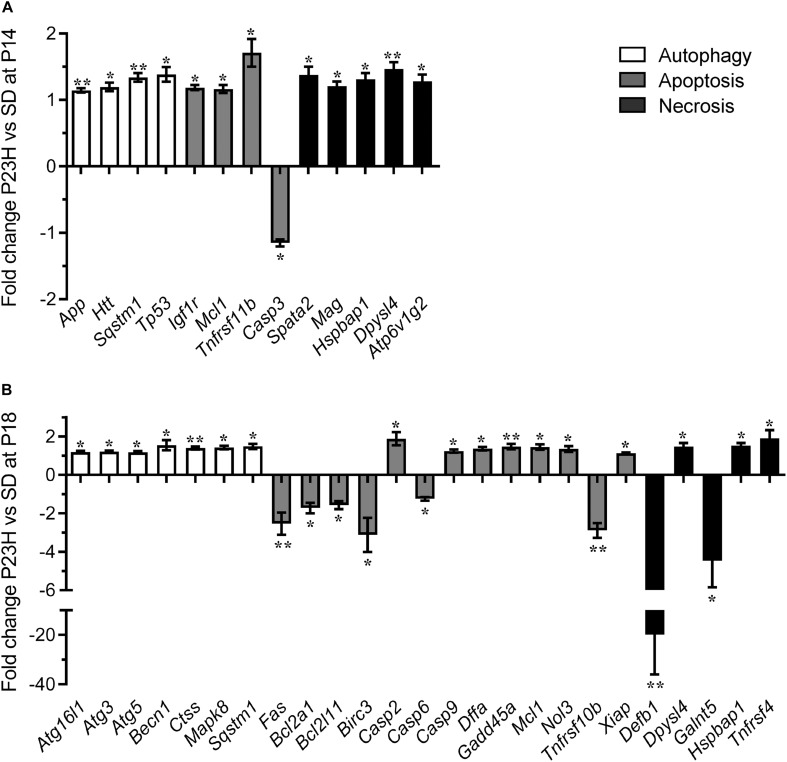
Dysregulation of programmed cell death-related genes in P23H-3 retinae. Changes in programmed cell death related genes (fold regulation) in P23H-3 compared to age-matched SD rats at P14 **(A)** and P18 **(B)**. Genes are clustered according to involvement in autophagy, apoptosis or necrosis pathways as defined by Qiagen. Data presented as mean ± SEM, Student’s *t*-test, *p < 0.05, **p < 0.01.

To investigate the mechanisms by which photoreceptors die in the P23H-3 retina, we analyzed gene expression changes in several cell death pathways using a commercial quantitative RT-PCR array system at P14 and P18. At P14, 13 of 84 genes on the PCR array showed significantly different levels of expression in P23H-3 compared to SD retinae (Figure 5A and Supplementary Table S3). The only down-regulated gene (−1.2 fold; *p* = 0.047) was the apoptotic effector, caspase-3. By contrast, three other genes implicated in the apoptotic pathways *Igf1r* (1.2 fold; *p* = 0.007), *Mcl1* (1.2 fold; *p* = 0.044), and *Tnfrsf11b* (1.7 fold; *p* = 0.017) were significantly up-regulated. Importantly, the master regulator of cell death, *Tp53* was also significantly up-regulated (1.4 fold; *p* = 0.022). The five genes that were broadly classified as being associated with necrosis (*Spata2*, *Mag*, *Hspbap1*, *Dpysl14*, and *Atp6v1g2*) were all up-regulated to similar levels (1.2 to 1.5-fold; *p* < 0.05). Similarly, three genes associated with autophagy (*App*, *Htt*, *Sqstm1*) were up-regulated (1.1 to 1.3-fold; *p* < 0.05).

At P18, 25 of 84 genes on the array showed significantly different levels of expression in P23H-3 compared to SD retinae (Figure 5B and Supplementary Table S5). Seven genes associated with the autophagy pathway (*Atg16l1*, *Atg3*, *Atg5*, *Becn1*, *Ctss*, *Mapk8*, *Sqstm1*) were significantly (*p* < 0.05) up-regulated at similarly low levels (1.2 to 1.5-fold). Of the twelve genes associated with the apoptotic pathway, six (*Fas*, *Bcl2a1*, *Bcl2l11*, *Birc3*, *Casp6*, *Tnfrsf10b*) were significantly down-regulated (−1.2 to −3.1-fold; *p* < 0.05) and seven (*Casp2*, *Casp9*, *Dffa*, *Gadd45a*, *Mcl1*, *Nol3*, *Xiap*) were significantly up-regulated (1.2 to 1.9-fold; *p* < 0.05). The largest expression changes were observed for two of the five differentially expressed necrosis genes (*Defb1*, *Galnt5*), which were significantly down-regulated −19.9 (*p* = 0.001) and −4.5-fold (*p* = 0.033), respectively. The other three necrosis-associated genes (*Dpysl4*, *Hspbap1*, *Tnfrsf4*) were significantly up-regulated 1.1 to 1.9-fold (*p* < 0.05).

The broad clustering of the dysregulated genes represented on the cell death array into the autophagy, apoptosis and necrosis cell death pathways, suggested that all three types of programmed cell death (Figure 5) are potentially modulated during P23H-mediated photoreceptor degeneration. To determine the activation states of the pathways, we conducted IPA analyses of this limited set of expression data using the Molecule Activity Predictor function in IPA. IPA predicted that the mitochondrial associated parts of the apoptotic pathway were inhibited at P14, but likely to be activated at P18 (Supplementary Figure S4). By contrast, the autophagy pathway appeared to be activated at both P14 and P18 (Supplementary Figure S5). While a canonical pathway for necrosis was not available in IPA, a network analyses incorporating key necroptosis genes, indicated that components of the necroptotic pathway, particularly RIPK1 and RIPK3 were predicted to become activated from P14 to P18 (Supplementary Figure S6).

To validate the mRNA analyses and to further discriminate which of these pathways were activated at P18, we conducted RPPA analyses on protein extracts from SD and P23H-3 retinae and immunofluorescence analyses on retinal sections at P18. Some genes showed significant changes at mRNA level (*Xiap*, *Mcl1*, *Bcl2l11*), which were not evident at the protein level due to these being below the threshold of detection (Table 1). Interestingly, while there appeared to be a significant increase in cleaved caspase-3, the levels were also below threshold for the antibody using this technique, suggesting that there was no large-scale induction of caspase-dependent apoptotic death pathways. While not all gene expression data could be validated by this technique, due to restricted antibody availability, the data indicated significantly reduced phosphorylation of AKT, which is an important regulator of apoptosis and autophagy (Heras-Sandoval et al., 2014), but also of necroptosis (Liu et al., 2014).

**TABLE 1:** Comparison of mRNA and protein expression of genes involved in cell death pathways in P18 retinae.

PCR Array			Reverse Phase Protein Array				
Array Pathway*	Symbol	FC	*p*-value	Protein	FR	*p*-value	Comment
Anti-apoptotic	*Igf1r*	–1.036	0.618	IGF1R	0.872	0.281	
Anti-apoptotic/Autophagy	*Akt1*	–1.017	0.790	AKT1-3 protein	0.973	0.787	
				AKT_P Thr308	0.804	0.039	
				AKT_P S473	0.561	0.042	
Autophagy	*Pten*	1.205	0.058	PTEN	0.909	0.247	
Autophagy	*Mapk8/Jnk2*	1.205	0.058	JNK2	0.875	0.210	At or below threshold
				JNK1/JNK2/JNK3	0.953	0.803	At or below threshold
Anti-apoptotic; Autophagy	*Bcl2*	–1.061	0.747	BCL-2	1.254	0.143	Below detection threshold
Anti-apoptotic	*Xiap*	1.122	0.037	XIAP	0.910	0.538	Below detection threshold
Pro- and Anti-apoptotic, Autophagy	*Bcl2l1/BclxL*	1.425	0.127	BCL-XL	1.030	0.639	
Anti-apoptotic	*Mcl1*	1.445	0.013	MCL1	1.932	0.223	Below detection threshold
Autophagy	*Rps6kb1*	1.291	0.321	RPS6KB1	1.375	0.134	Below detection threshold
Pro-apoptotic	*Bcl2l11/Bim*	–1.581	0.020	BIM	1.206	0.476	Below detection threshold
Pro-apoptotic	*Tp53*	–1.363	0.053	TP53	1.080	0.294	
				TP53_P S15	3.170	0.061	Below detection threshold
Pro-apoptotic; Autophagy	*Bax*	–1.054	0.690	BAX	1.358	0.085	
Pro-apoptotic; Autophagy	*Casp3*	1.188	0.061	CASP3	0.898	0.270	
				CASP3_Cleaved Asp175	1.828	0.022	Below detection threshold
Pro-apoptotic	*Casp7*	–1.079	0.881	CASP7_Cleaved Asp198	1.475	0.064	At or below threshold
Necrosis	*Parp1*	–1.136	0.573	PARP	0.972	0.852	

Significant *p*-values are highlighted in gray.Abbreviations: *, as supplied by Qiagen; FC, fold-change; FR, fold-regulation.

### Photoreceptor Death in P23H-3 Does Not Involve Mitochondrial-Mediated Pathways

**FIGURE 6:**
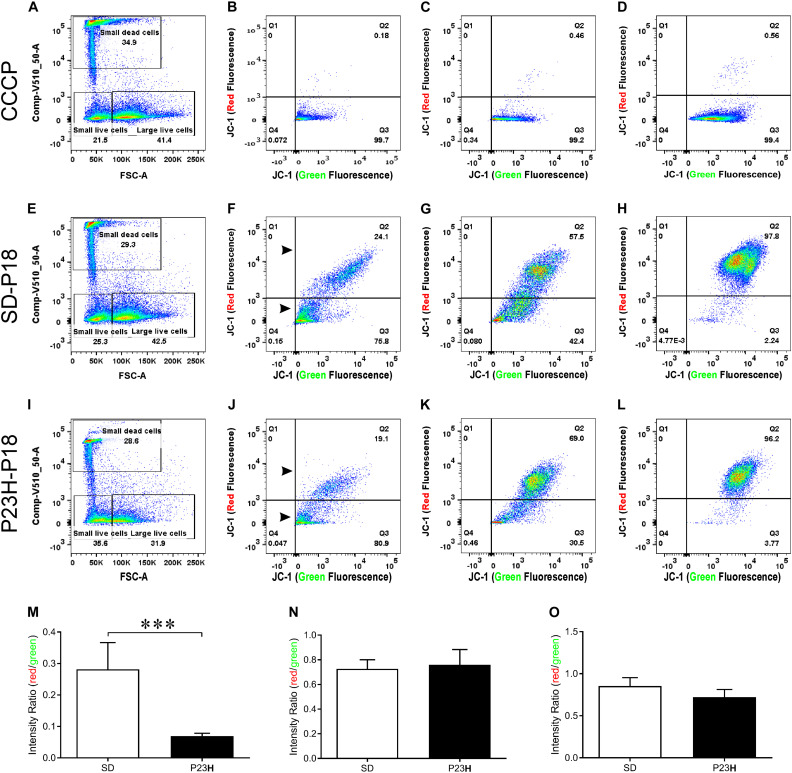
Mitochondrial membrane potential (Δψm) in P23H-3 and SD retinal cells at P18. Cell gating strategy for the analysis of retinal cells with different mitochondrial membrane potentials **(A,E,I)** was based on cell size and DAPI fluorescence. Three distinct subpopulations of cells were detected in all samples: small dead cells **(B,F,J)**, small live cells **(C,G,K)**, and large live cells **(D,H,L)**. Representative plots for each of the three populations of cells show levels of polarized (red fluorescence) and depolarized (green fluorescence) mitochondria due to the JC1 dye, in CCCP-treated **(B–D)**, SD **(F–H)**, and P23H-3 **(J–L)** retinal cells. While no major fluorescence shifts could be detected for small live and large live cells between SD and P23H-3 samples, there appeared to be a slight shift from red- green for the small dead cells (Arrowheads; **F,J**). Quantification of red:green fluorescence intensity ratio in the three cell populations from P23H-3 and SD samples showed that small dead **(M)**, but not small live **(N)** or large live cells **(O)**, had a significant drop in red:green fluorescence intensity ratio (*p* < 0.0001; two-tailed unpaired *t*-test) in the P23H-3 retinae. Data presented as mean ± SEM, Student’s *t*-test, ***p < 0.001, *n* = 6 in each group.

To investigate the involvement of mitochondrial-mediated cell death in photoreceptor degeneration in P23H-3, we utilized a flow cytometric JC-1 assay to investigate the mitochondrial membrane potential (Δψm) in P23H-3 and SD retinae at P14 and P18 (Reers et al., 1995; Salvioli et al., 1998). The JC-1 assay is based on a shift in emission fluorescence from red to green wavelengths if the mitochondrial membrane is depolarized. The gating strategy (Supplementary Figure S3) allowed us to distinguish three different cell populations based on cell size and cell viability (Figure 6A).

Overall, similar proportions of cells were isolated for each population in each group (Figures 6A,E,I). While previous studies, using a *Nrl-GFP* transgene in mice had indicated that rod photoreceptors are the smallest cells isolated by flow cytometry (Akimoto et al., 2006), our quantitative PCR analyses of the cell populations we isolated, indicated that only dead small cells were enriched for rod photoreceptors compared to the other populations, therefore we also analyzed this cell population. The positive control cells, involving treatment of cells with carbonyl cyanide m-chlorophenyl hydrazone (CCCP), clearly showed a marked shift from red to green fluorescence (Figures 6B–D), indicating drug-induced mitochondrial membrane depolarization. No such fluorescence shift was detected in small live (Figure 6K) or large live cell (Figure 6L) populations of the P23H-3 retinal cells, compared to SD control retinal cells (Figures 6G,H). In the small dead cell populations, there appeared to be an increase in the number of small cells with higher levels of green fluorescence (compare arrowheads, Figures 6F,J). Quantification of the ratio of red:green fluorescence intensity for all three cell populations (Figures 6M–O) showed that only small dead cells were significantly different (*p* < 0.0001, two-tailed unpaired *t*-test) in P23H-3 compared to SD retinae (Figure 6M). To determine if this feature was unique to P18 retinae, we repeated the analysis at P14 and obtained similar data (Supplementary Figure S4).

**FIGURE 7:**
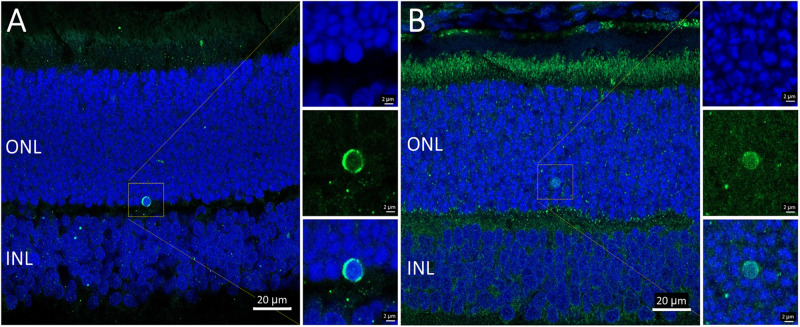
Activated caspase-3 and AIF staining in P23H-3 retinae at P18. Confocal images of P18 retinal sections labeled with anti-cleaved caspase-3 (**A**, green) and AIF (**B**, green), showing only occasional cells were labeled. AIF is clearly detectable in the numerous mitochondria in the photoreceptor inner segments and also in the retinal pigmented epithelium and outer plexiform layer. Each of the labeled cells is shown at higher magnification in the three insets showing fluorescence due to DAPI (blue), the antibody (green) and the merged image. Abbreviations: ONL, outer nuclear layer; INL, inner nuclear layer. Scale bar = 20 μm **(A,B)**; 2 μm (insets).

Overall, the flow cytometry data indicated that in small or large live cells, isolated from the P23H-3 retina, there was no evidence of significant changes in mitochondrial permeability. However, greater numbers of small dead cells, isolated from the P23H-3 retina had disrupted mitochondrial permeability. Quantitative PCR analyses of P23H-3 retinae at P16 showed that this population of cells was enriched for rod photoreceptors by approximately 1.6- to 1.8-fold (Supplementary Table S1). These data suggested that dying or dead photoreceptors were undergoing or had undergone increased mitochondrial permeability. Alternatively, this population of cells may arise as an artifactual effect of the isolation protocol or represent dead cells (including photoreceptors) at the time of collection and isolation. Thus, this could be an artifactual apparent increase in mitochondrial permeability as a consequence and not a cause of cell death. To further investigate the involvement of mitochondrial-mediated cell death mechanism, we conducted immunostaining for cleaved caspase-3 and nuclear AIF at P18. Only occasional photoreceptor cells in the ONL of P23H-3 were positive for either cleaved caspase-3 or AIF at P18 (Figures 7A,B, respectively). Similarly, only occasional stained cells were detected in the SD retinae (not shown). These observations, combined with the flow cytometric JC-1 assay, suggest that classical caspase-mediated apoptosis (either extrinsic or intrinsic) or caspase-independent apoptosis via AIF translocation to the nucleus are not major contributors to photoreceptor death in the P23H-3 retina. Consistent with this, expression and activation of proteins associated with apoptosis were poorly detectable (BCL-2, XIAP, MCL1, BIM, TP53, cleaved CASP3) or did not show significant changes (IGFR1, BCL-XL, BAX, CASP3) in retinal extracts by RPPA analysis (Table 1).

### Evidence for Increased Autophagy During Photoreceptor Cell Death in P23H-3 Retina

**FIGURE 8:**
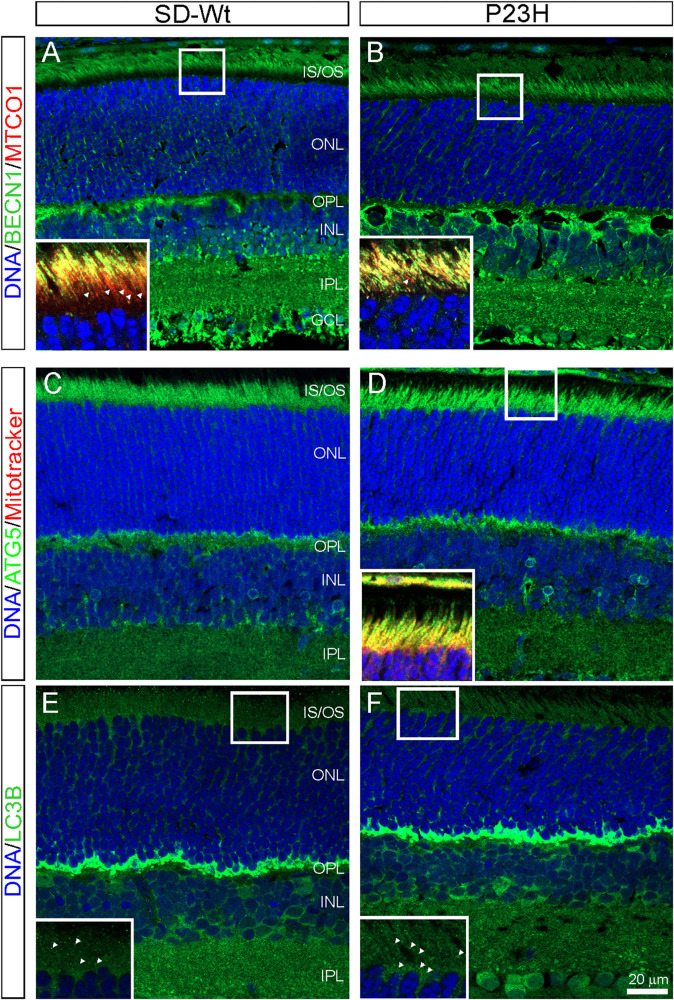
Autophagy markers (BECN1, ATG5, LC3B) in P23H-3 retinae at P18. Confocal images of P18 retinal sections labeled with BECN1 (**A,B**, green) and cytochrome oxidase (MTCO1; insets **A,B**, red), ATG5 (**C,D**, green) and MitoTracker (inset, **D**) and LC3B (**E,F**, green) in wild-type SD **(A,C,E)** and P23H-3 **(B,D,F)** retinae. Staining for BECN1 **(B)** and ATG5 **(D)** appeared to be mildly increased and more distinct in the P23H-3 retina. The yellow colored co-labeling with cytochrome oxidase (insets **A,B**, red) or MitoTracker (inset, **D**, red) indicates increased association of BECN1 and ATG5 with mitochondria in P23H inner segments. Labeling of phagosomes (arrowheads) in the inner segments with LC3B appeared to be increased in the P23H-3 **(F)** compared to SD retina **(E)**. All sections were counter-stained with DAPI (blue) to label cell nuclei. Abbreviations: GCL, ganglion cell layer; INL: inner nuclear layer; IPL, inner plexiform layer; IS/OS, inner/outer segments; ONL, outer nuclear layer; OPL, outer plexiform layer. Scale bar: **(A–F)**, 20 μm; insets, 10 μm.

The PCR array data indicated moderate increases in autophagy-related proteins in P23H-3 retinae at both P14 and P18. The involvement of autophagy in photoreceptor death was examined by immunofluorescence of BECN1 and ATG5. The staining for BECN1 (compare Figures 8A,B) and ATG5 (compare Figures 8C,D) were relatively diffuse in the SD inner segments but appeared brighter and more distinct in the inner segments of the P23H-3 photoreceptors and the OPL. As translocation of BECN1 to mitochondria is associated with autophagosome formation and mitophagy (Gelmetti et al., 2017), we co-labeled sections with cytochrome oxidase (CO) and found increased association of BECN1 with mitochondrial CO in the proximal region of the inner segments (insets Figures 8A,B). Similar results were obtained when ATG5 was co-localized with MitoTracker (inset Figure 8D). The increased association of BECN1 and ATG5 with mitochondria suggests increased mitophagy in the inner segments of the P23H-3 retina. Consistent with this, there appeared to be moderately increased phagosome formation as detected by immunofluorescence with an antibody to LC3B (Figures 8E,F insets).

### Widespread Necroptosis Is a Major Contributor to P23H-3 Photoreceptor Cell Death

**FIGURE 9:**
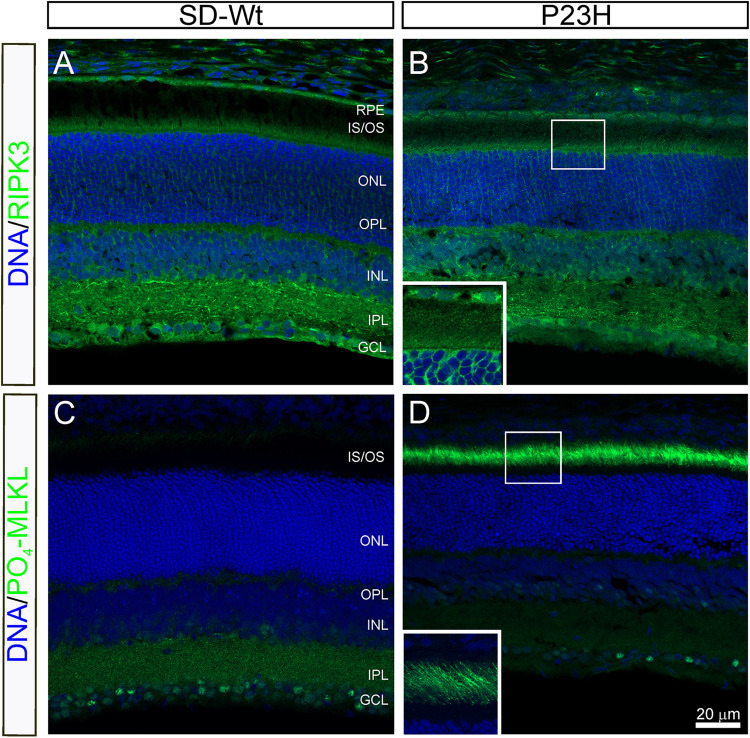
RIPK3 and phospho-MLKL staining in P23H-3 retinae at P18. Confocal images of P18 retinal sections labeled with RIPK3 (**A,B** green) and phosphorylated (S345)-MLKL (**C,D** green) antibodies. No differences were noted in localization of the RIPK3 kinase in the wild-type SD **(A)** compared to P23H-3 **(B)** retina, with weak but specific staining observed in the inner segments of the photoreceptors (inset, **B**) as well as in the OPL, INL, IPL, and GCL. By contrast, there was a marked difference in the expression of phosphorylated MLKL, with dramatic induction in the outer segments of the P23H-3 retina **(D)**, which was completely absent in the wild-type SD retina **(C)**. The inset in panel **(D)**, shows the distinct localization in the outer segments. Abbreviations: GCL, ganglion cell layer; INL: inner nuclear layer; IPL, inner plexiform layer; IS/OS, inner/outer segments; ONL, outer nuclear layer; OPL, outer plexiform layer; RPE, retinal pigmented epithelium. Scale bar = 20 μm.

From P14 to P18, the most dramatic changes in gene expression occurred in genes associated with necrosis (Figure 5) and combined with the altered activation of AKT, shown by RPPA, suggesting the induction of necroptosis. To investigate the possibility of programmed necrosis (necroptosis) being involved in photoreceptor cell death, we examined the localization of the kinase, RIPK3, and the phosphorylated form of its target, MLKL. While there appeared to be no difference in the expression of RIPK3 in the inner segments of P23H-3 retinae (Figure 9B) compared to the SD control retinae (Figure 9A), expression of phospho-MLKL was markedly increased in the inner and outer segments of the P23H-3 retinae (Figure 9D) compared to wild-type SD retinae (Figure 9C). These data suggest that at P18, when photoreceptors are actively dying, the necroptosis pathway is highly activated.

## Discussion

The present study provides a detailed and comprehensive phenotypic analysis of retinal structure and function in the P23H-3 rat model of inherited retinal degeneration and highlights the complexity of mechanisms involved in photoreceptor death. Notably, mechanisms associated with necroptosis are particularly important for rod photoreceptor death.

### P23H-3 Rats Show a Progressive Decline in Retinal Function From P30

In wild-type SD rats there was a clear developmental profile for rod ERG responses as reported previously (Fulton et al., 1995; Gibson et al., 2013; Nadal-Nicolas et al., 2018), with significant increases in amplitudes of a- and b-waves and OP from P18 to P30 with subsequent decreases in amplitudes at P60 and P90. A similar developmental pattern of ERG responses was seen for the cone b-wave response, which was significantly increased at P30 from P18 in SD normal rats, with a subsequent decline at P60. The functional increases from P18 to P30 likely reflect the postnatal development of the photoreceptors, including elongation of outer segments, increased expression of opsins (Fulton et al., 1995; Chrysostomou et al., 2009; Gibson et al., 2013) as well as continued functional and morphological modifications of synaptic connections of downstream circuits (Tian, 2004, 2008). By contrast, P23H-3 rats showed significantly attenuated amplitudes of rod photoreceptor (a-wave) and post-receptoral responses (b-wave and OP) as early as P30. At P60, the amplitude of the rod a-wave, b-wave and OP decreased by 60, 40, and 62%, respectively and are similar to the reductions in rod a-wave b-wave amplitudes reported previously (Machida et al., 2000; Chrysostomou et al., 2009; LaVail et al., 2018). However, the normal developmental functional overlay raises the question of whether these reductions in ERG components after P30 are due to impairment of the normal functional maturation of the retina and/or the underlying P23H-induced cell loss.

Comparison of the mean percentage loss of rod a-wave, b-wave and OP amplitudes from P30-P90 showed that while the percentage decline in the summed OP was significantly greater than the percentage decline in the a-wave, the rate of decline over the 3 months was similar to that of the declines in the a-wave, suggesting amacrine cell function declines in parallel with the rod photoreceptors. However, the percentage decline in the b-wave plateaued at P90, suggesting that Müller/bipolar cell function is somewhat preserved at this stage.

Our analysis of cone post-photoreceptoral responses indicated that P23H-3 animals had significantly lower cone b-wave amplitudes compared to SD controls at P30 and P60, while the cone, OP amplitudes remained relatively unaffected. This suggests that cone dysfunction occurs from an early stage of disease – earlier than has been previously reported (Machida et al., 2000; Chrysostomou et al., 2009). While previous studies showed significantly lower numbers of L/M-cones in P23H-1 homozygous albino rat retinae when compared to SD rats from P30 to P180 (Garcia-Ayuso et al., 2013), we did not find any significant differences in total cone numbers detected by PNA in the P23H-3 retina at P30 (Supplementary Figure S2G). Overall, these data suggest that in the P23H-3 strain, cone dysfunction, but not cone death, occurs from P30 or even earlier. However, it should be noted that the analyses in this study were conducted on albino P23H-3 rats and that the timing of retinal functional and morphological changes are different when this mutation is present on a pigmented strain background (Cuenca et al., 2014; Lowe et al., 2019). Indeed, it has been proposed that the lack of pigmentation possibly affects cone function more than rod function over time (Cuenca et al., 2014).

### Structural Changes and Patterns of Cell Loss Follow Functional Losses

While fundus imaging in these albino rats failed to reveal overt characteristics of RP (due to lack of pigmentation), both OCT and histological analyses demonstrated significant, progressive thinning of ONL in P23H-3 rats from P60 onward, reaching 22% reduction at P90. These observations are consistent with previous studies indicating similar progression of retinal thinning, with 15% loss of photoreceptors by P30, 50% by P203 (Machida et al., 2000) and 68% by P230 (Chrysostomou et al., 2009). While our OCT measurements did not show significant changes in the photoreceptor inner/outer segments, histological studies have detected shortened OS from P30 to P203 (Machida et al., 2000), suggesting that OCT resolution may not be sufficient to detect subtle structural changes in rat eyes.

Our TUNEL assays show prolific photoreceptor cell death by P18 that gradually subsides from P30 to P90. Consistent with previous studies, these results suggest that retinal degeneration is biphasic, with a rapid initial phase, with a peak occurring at approximately P17-P20 (Lee et al., 2003; Yu et al., 2004; Kaur et al., 2011; Zhu et al., 2013), followed by a slower phase up to P60. In particular, the early phase of photoreceptor degeneration overlaps with naturally occurring morphogenetic death of photoreceptors during retinal maturation and is a critical period when photoreceptors are susceptible to oxygen levels (Maslim et al., 1997). As a result, cells may be even more susceptible to stress-induced damage by *Rho* mutations during this period (Lee et al., 2003).

Quantification of ONL thickness across retinal eccentricity indicated that by P60 there was significantly greater loss of ONL cells in the central and mid-peripheral regions. Consistent with this, TUNEL analyses showed greater numbers of TUNEL^+^ cells in these regions at P30. This is consistent with a recent study reporting higher rate of retinal degeneration in the superior than inferior hemisphere of P23-3 strains from P90 onward and that the loss of photoreceptors proceeds in a central-peripheral gradient (LaVail et al., 2018). By contrast Machida et al. (2000), using resin-embedded sections, found no significant differences in ONL cell counts in superior and inferior hemispheres of P23H-3 retinae from 4 to 29 weeks of age (Machida et al., 2000). While the reasons for this discrepancy in the P23H-3 rat retina are not clear, in human patients with P23H mutations, degeneration usually initiates in the mid peripheral retina and progresses gradually to the macula and more peripheral regions, with loss of rod photoreceptors in the far periphery occurring at relatively advanced stages (Heckenlively et al., 1991; Stone et al., 1991).

### Multiple Death Pathways Are Modulated During Photoreceptor Death

While early studies, based on TUNEL assays, suggested photoreceptor cell death was via an apoptotic mechanism (Chang et al., 1993), TUNEL also labels DNA fragments generated by non-apoptotic mechanisms of cell death, such as necrosis (Grasl-Kraupp et al., 1995). Subsequently, various different mechanisms of cell death have been proposed to mediate photoreceptor cell death in the degenerating retinae of various animal models for RP. These include intrinsic and stress-induced, caspase-dependent apoptosis (Jomary et al., 2001), as well as activation of calpains and AIF-dependent photoreceptor cell death, also known as “parthenatos” (Paquet-Durand et al., 2010; Kaur et al., 2011; Comitato et al., 2016). It has been shown that the mutant P23H protein undergoes abnormal folding and its accumulation leads to ER stress and the UPR (Lin et al., 2007). Components of the three UPR response pathways are elevated at P15 (Kroeger et al., 2012), P30 (Lin et al., 2007; Gorbatyuk et al., 2010), and at P60 (Kroeger et al., 2012), correlating with the onset of receptor death, functional loss and structural decrements, respectively. While short term activation of UPR facilitates degradation of accumulated unfolded proteins via the proteasome or by autophagy, prolonged UPR can lead to activation of apoptosis or, as more recent evidence suggests, necroptosis (Iurlaro and Munoz-Pinedo, 2016; Wang et al., 2018). Thus, UPR can be an early protective response or, if sustained, a mechanism that contributes to photoreceptor death.

To gain further insight into molecular pathways downstream of this UPR that potentially mediate P23H-RHO related photoreceptor death, we used quantitative RT-PCR arrays, focused on genes that are involved in major cell death pathways (apoptosis, autophagy, and necrosis), to survey expression changes at P14 and P18. Pathway analysis of the expression data indicated that while both autophagy and necroptosis are activated at both P14 and P18, activation of the intrinsic apoptotic pathway was predicted to occur only at P18.

### Photoreceptor Death Does Not Involve Apoptosis or Parthenatos

Overall, the mildly dysregulated expression changes at P14 are suggestive of mechanisms that suppress apoptosis, but promote cell survival, autophagy and necroptotic pathways. For example, *Tnfrsf11b*, a TNF superfamily receptor involved in bone regulation, has been detected in vitreous, retina and RPE (Ehlken et al., 2015) and can inhibit apoptosis mediated via the P38 MAPK stress pathway (Holen et al., 2005). Similarly, *Mcl1* is a member of the Bcl2 family of anti-apoptotic proteins and the longest form of its three alternatively spliced variants enhances cell survival (Morciano et al., 2016). Finally, *Igfr1*, is commonly considered as anti-apoptotic and through its binding of insulin and IGF1 can potentially rescue retinal neurons from cell death by activating PI3K/AKT/mTOR cell survival pathway (Seigel et al., 2000; Barber et al., 2001; Punzo et al., 2009), which in turn can modulate autophagy and necroptosis.

Similarly, the expression patterns of apoptotic pathway genes at P18 suggest contrasting regulatory effects on pathway activity. The dysregulation of some genes (e.g., *Bcl2l11*, *Fas*, *Casp6*, *Gadd45a*, *Mcl1*, *Nol3*, *Tnfrsf10b*, *Xiap*) potentially inhibit apoptosis whereas dysregulation of others (e.g., *Birc3*, *Bcl2a1*, *Casp2*, *Casp9*, *Dffa*, *Gadd45a*) potentially promote apoptosis. In addition, the dysregulation of other apoptotic genes is suggestive of links to necroptosis (via CASP2 interaction with RIPK1 complexes) and to autophagy (via MCL1 phosphorylation by GSK3beta/BECN1) (Bouchier-Hayes and Green, 2012).

Consistent with these expression findings of variable activation of intrinsic apoptosis, our JC-1 dye in flow cytometry assays of mitochondrial permeability at P14 and P18, demonstrated very little evidence for changes in mitochondrial permeability at P14 and P18 in live cells collected from P23H-3 retinae. It is unclear if the significant changes in mitochondrial permeability in small dead cells reflects a greater susceptibility of P23H-3 retinal cells to undergo apoptotic cell death due to the dissociation procedure, or if more dead cells are present in these retinae than the wild-type at the time of collection. However, consistent with these mitochondrial permeability data, immunolabelling for cleaved caspase-3 as a marker for intrinsic/extrinsic apoptosis and nuclear AIF for non-caspase mediated parthenatos showed only sparse reactivity in the ONL of P23H-3 retinae at P18. Similar findings of negligible caspase-3 activity have been reported in the P23H-1 strain at P15 (Kaur et al., 2011; Arango-Gonzalez et al., 2014). Nor is there evidence for caspase-9 activity, cytochrome-C leakage or abundant nuclear translocation of cleaved AIF in P23H-1 rats (Kaur et al., 2011; Sizova et al., 2014). Overall, these studies indicate that cell death mechanisms, acting via altered mitochondrial permeability via either caspase-dependent or caspase-independent parthenatos pathways, are not major contributors to cell death induced by P23H RHO.

### Increased Autophagy During Photoreceptor Death in P23H-3 Retina

The upregulation of several genes associated with autophagy (*App*, *Htt*, *Sqstm1*, and *Tp53*) at P14 can be interpreted as both cytoprotective and potentially increasing autophagy. For instance, while pathological processing of amyloid precursor protein (APP) to amyloid β in Alzheimer’s disease has been implicated in disrupting autophagy via the BECN1 complex (Salminen et al., 2013), the normal non-amyloidogenic processing of APP has been associated with neuroprotection in the retina (Cimdins et al., 2019).

Similarly, at P18 there is marked dysregulation of genes that are core components of the autophagy machinery (*Atg16l1*, *Atg3*, *Atg5*, and *Becn1*) (Suzuki et al., 2017) and also *Sqstm1*, which is a key mediator in marking and directing cellular components to the autophagy machinery (Lamark et al., 2017). These data are consistent with a previous study showing dynamic expression profiles of autophagy-related genes in P23H-3 retinae at P21 (Sizova et al., 2014). Moreover, autophagy and lysosomal proteins have been shown to colocalize with mutant P23H protein (Kaushal, 2006). In our experiments, ATG5 and BECN1 showed increased co-localization with mitochondria in the inner segments of the mutant photoreceptors, suggesting that there is increased mitophagy. Indeed, localization of BECN 1 at mitochondrial membranes has been shown to promote tethering of ER and mitochondria to initiate autophagosome formation (Gelmetti et al., 2017).

Overall, the upregulation of these autophagy-promoting genes is consistent with the notion that ER stress, induced by accumulating misfolded P23H rhodopsin protein, induces an autophagic response in mice carrying the P23H mutation (Song et al., 2018; Yao et al., 2018). However, somewhat surprisingly inhibition of autophagy increases photoreceptor survival, whereas increasing autophagy promotes retinal degeneration, suggesting that autophagy actually contributes to PR cell death (Yao et al., 2018). Thus, while autophagy may help to clear ER-resident misfolded proteins, it likely comes at the cost of increased autophagy-mediated loss of the ER and mitochondria with consequent photoreceptor cell death.

### Necroptosis Is a Major Contributor to Photoreceptor Death in P23H-3 Retina

Various genes that were up-regulated at P14 and P18 suggest that there was activation of necroptosis in the P23H-3 retina. The well-known tumor suppressor, *p53* (TP53), has been associated with not only caspase-dependent and caspase-independent pathways of apoptosis, but also with non-apoptotic mechanisms of cell death, including programmed necrosis (necroptosis) (Nikoletopoulou et al., 2013; Ranjan and Iwakuma, 2016). The coincident up-regulated expression of *Dpysl4*, a direct target of P53 transcriptional activity and calpain cleavage activity (Kimura et al., 2011), suggests that, in the retina at P14, P53 may upregulate a calpain-mediated necrotic cell death pathway. Further evidence for upregulation of necrosis comes from the increased expression of genes that have been implicated in programmed necrotic cell death (*Mag*, *Spata2*, *Hspbap1*, and *Atp6v1g2*). In particular, upregulation of *Spata2*, which modulates TNFα signaling, can promote TNFα-mediated necroptosis and inhibit the apoptotic pathway (Schlicher et al., 2017). Indeed, inhibition of apoptosis is a common contributing trigger for induction of necroptosis (Morris et al., 2018). Similarly, at P18, dysregulation of *Casp2* can potentially activate necroptosis via interaction with RIPK1 complexes (Bouchier-Hayes and Green, 2012).

It has been suggested previously that necroptotic-mediated signaling is an important driver of rod photoreceptor cell death in the P23H-3 model, whereas NLRP3 inflammasome activation is the mechanism for subsequent cone cell death Viringipurampeer et al., 2019. Viringipurampeer et al. (2019) demonstrated increased expression of RIPK3 in P23H-3 rats by western blot at P21, P45 and P120 and its co-localization with ID4 a rhodopsin marker. Moreover, long-term injections of a combination of inhibitors for RIPK1, PARP and calpain with an anti-oxidant/inflammatory inhibitor ameliorated ONL cell/outer segment loss and provided some protective effect on cone flicker amplitudes (Viringipurampeer et al., 2019). While our study did not demonstrate any detectable change in RIPK3 expression or localization in the P23H-3 photoreceptor outer segments, we did demonstrate dramatically increased localization in the outer segments of phosphorylated MLKL, which is the substrate for the active RIPK1/RIPK3 complex and, in its phosphorylated form, a key effector of necroptosis (Shan et al., 2018).

## Conclusion

In conclusion, we have shown that in P23H-3 rats various cell death pathways are modulated from P14 to P18, with distinct activation of autophagy and necroptosis but limited activation of caspase-dependent or caspase-independent (parthenatos) pathways. The induction of MLKL phosphorylation at P18 provides definitive evidence for large-scale activation of necroptosis as the principal cause of photoreceptor death in the P23H-3 retina. This cell death manifests as a functional decline in rod photoreceptor, bipolar and amacrine cell responses from P30 and morphologically as ONL cell losses from P60, which progresses from central to peripheral regions of the retina. In addition, we have documented an earlier onset of cone photoreceptor functional decline from P30 than described previously. Together with other studies, these data suggest that retinal degenerations may activate multiple cell death pathways (Lohr et al., 2006; Comitato et al., 2016; Viringipurampeer et al., 2016, 2019) and that therapies aimed at ameliorating photoreceptor death may need to target multiple cell death pathways as well as potential sequelae, such as inflammation, gliosis and microglial activation that can contribute subsequently to photoreceptor degeneration.

## Data Availability Statement

All datasets presented in this study are included in the article/Supplementary Material.

## Ethics Statement

The animal studies were reviewed and approved (AEC#1312958 and #1614030) by the Animal Ethics Committee of the University of Melbourne.

## Author Contributions

EF, AJ, and RI: conceptualization and supervision. KK, UG, EF, KV, and AJ: methodology. RI: software (IPA). KK and RI: validation, writing – original draft preparation, and visualization. KK, UG, AJ, and RI: investigation. EF: resources, project administration, and funding acquisition. KK, AJ, EF, and RI: data curation. All authors contributed to formal analysis and writing – review and editing.

## Conflict of Interest

The authors declare that the research was conducted in the absence of any commercial or financial relationships that could be construed as a potential conflict of interest.

## Footnotes

1 https://sph.uth.edu/retnet/

## Supplementary Material

Supplementary Figure 1 Measures of rod sensitivity, rod implicit time and OP implicit times by ERG in P23H-3 rats.

Supplementary Figure 2 Photoreceptor cell death and ONL morphometry in P23H-3 retina at different eccentricities and ages.

Supplementary Figure 3 Gating strategy for distinguishing different retinal cell populations, based on size and viability at time of isolation.

Supplementary Figure 4 Ingenuity pathway modeling of apoptosis gene expression data.

Supplementary Figure 5 Ingenuity pathway modeling of autophagy gene expression data.

Supplementary Figure 6 Ingenuity pathway modeling of necrosis gene expression data.

Supplementary Figure 7 Mitochondrial membrane potential (Δψm) in P23H-3 and SD retinal cells at P14.

Supplementary Table 1 Quantitative PCR analyses of flow cytometry separated cell populations.

Supplementary Table 2 Antibodies and fixation conditions used in immunofluorescence.

Supplementary Table 3 Antibodies used in reverse-phase protein arrays (RPPA).

Supplementary Table 4 Quantitative RT-PCR array data for cell death genes expressed in SD and P23H-3 rat retinae at P14.

Supplementary Table 5 Quantitative RT-PCR array data for cell death genes expressed SD and P23H-3 rat retinae at P18.
