# Supplementary material for: Photoreceptor Degeneration in Pro23His Transgenic Rats (Line 3) Involves Autophagic and Necroptotic Mechanisms
Source: Front Neurosci. 2020 Nov 3;14:581579. doi: 10.3389/fnins.2020.581579 (PMC7670078; doi:10.3389/fnins.2020.581579)
Supplement: Supplementary Figure 1 — Measures of rod sensitivity, rod implicit time and OP implicit times by ERG in P23H-3 rats. [file Data_Sheet_1.docx]

Supplementary Material

**
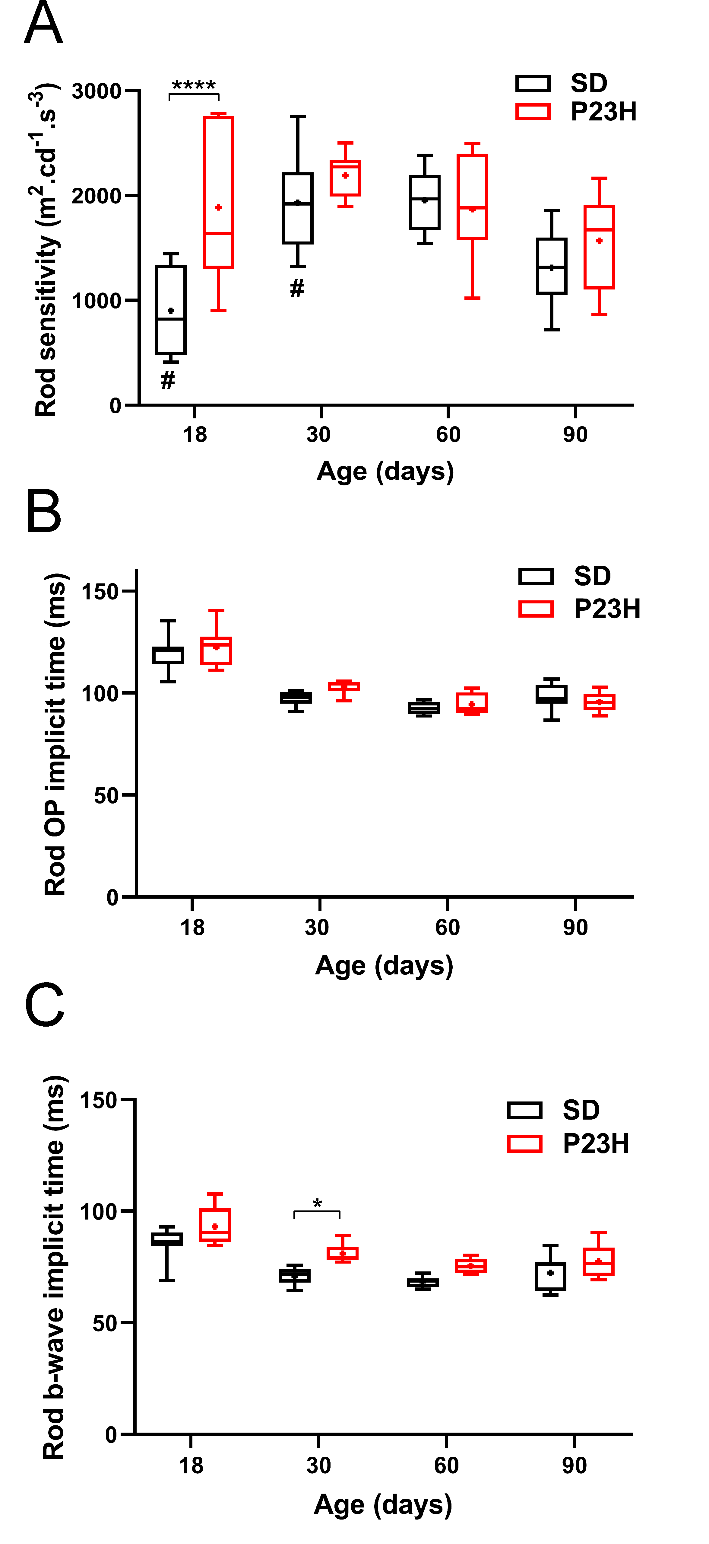
**

**Supplementary Figure S1.** **Box and whisker plots of rod sensitivity, rod implicit time and OP implicit times by ERG in P23H-3 rats. A**. The rod PIII (a-wave) sensitivity was significantly greater at P18 in the P23H-3 (p < 0.0001) compared to the SD wild-type retina but was similar to the SD thereafter. In the SD rats from P18 to P30, there was a significant increase in rod sensitivity (p < 0.001), suggestive of the development-related increase in photoreceptor sensitivity at this age[1]. **B**. No significant differences in rod implicit times were detected at any age. **C**. While the rod b-wave implicit times in P23H-3 appeared greater at each age, it was only significantly increased at P30 (p < 0.05). Overall, these findings are consistent with the findings of Machida et al., (2000) [2], who found that phototransduction sensitivity was not affected in P23H-3 rats at any age examined from P30 to P203. All box and whisker plots show interquartile range (box), median (transverse line), mean (+) and 95% confidence intervals (error bars); n ≥ 10 in each group.
