# Supplementary material for: Photoreceptor Degeneration in Pro23His Transgenic Rats (Line 3) Involves Autophagic and Necroptotic Mechanisms
Source: Front Neurosci. 2020 Nov 3;14:581579. doi: 10.3389/fnins.2020.581579 (PMC7670078; doi:10.3389/fnins.2020.581579)
Supplement: Supplementary Figure 2 — Photoreceptor cell death and ONL morphometry in P23H-3 retina at different eccentricities and ages. [file Data_Sheet_2.docx]

Supplementary Material


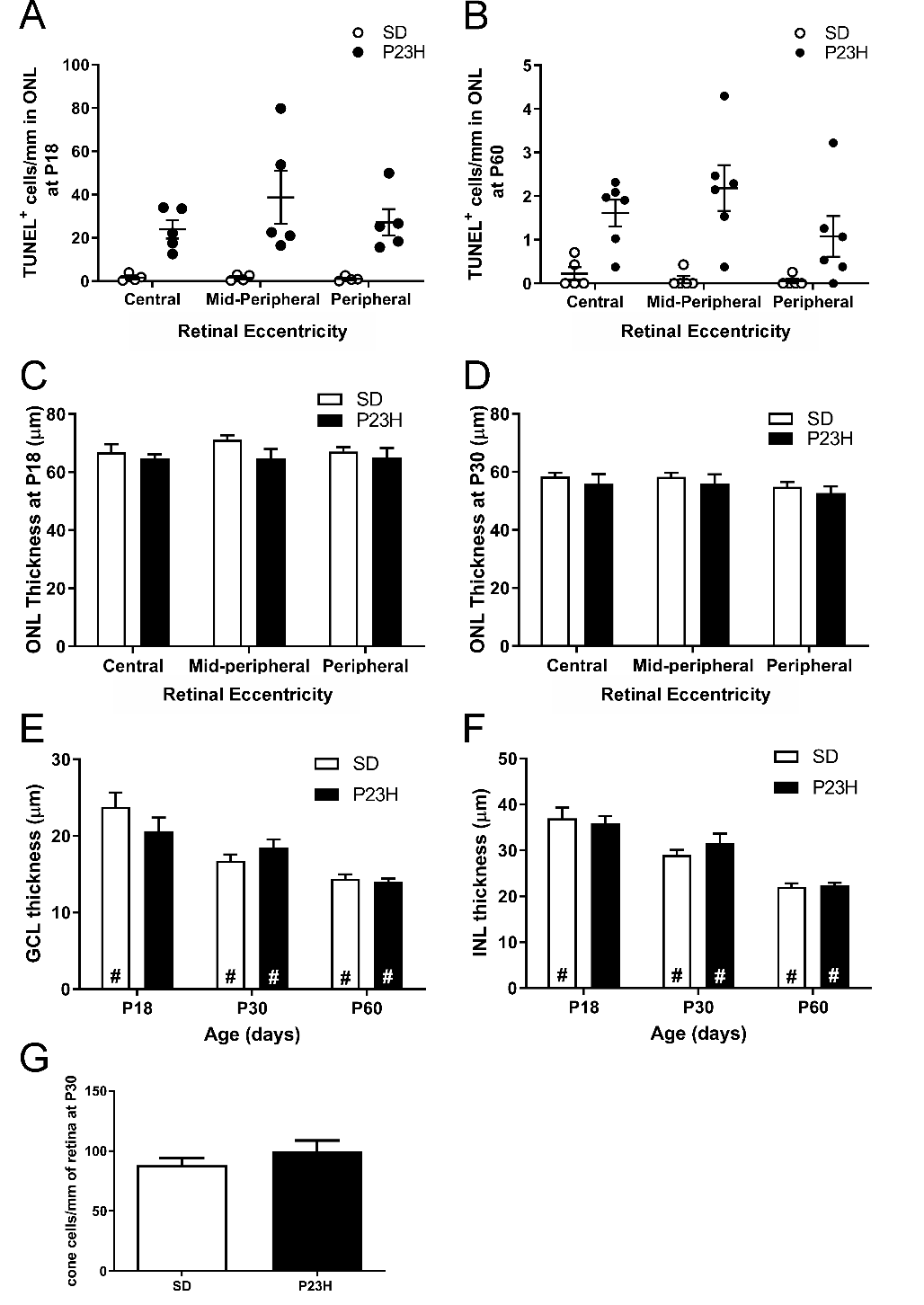


**Supplementary Figure S2. Photoreceptor cell death and retinal morphometry in P23H-3 retina at different eccentricities and ages**. **A-B.** Quantification of TUNEL^+^ cells in different regions of the ONL in P18 (**A**) and P60 (**B**) rats. As expected, no significant numbers of TUNEL^+^ cells were detected in the SD retinae at any age or eccentricity. At P18 (**A**), uniformly high mean numbers of TUNEL^+^ cells were detected in the central, mid-peripheral and peripheral regions of the retina, with no significant difference in the number of TUNEL^+^ cells at different eccentricities. By contrast, fewer TUNEL^+^ cells were detected at P60 and no significant differences were detected at different eccentricities (**B**). **C-D.** Quantification of ONL thickness in different regions of the ONL in P18 (**C**) and P30 (**D**) P23H-3 and SD rats by morphometric analyses of sections. No significant differences in ONL thickness were detected any eccentricity at P18 or P30. Data shown as mean ± SEM, n ≥ 5 in each group. **E-F.** Quantification of inner retinal layer (GCL, INL) thicknesses in P23H-3 and SD rats at P18, P30 and P60 show significant age-related decreases (#) in thickness but no difference between strains at any age. **G.** Quantification of cone cells, labeled with PNA, revealed no significant differences between SD and P23H-3 retinae at P30.
