# Supplementary material for: Photoreceptor Degeneration in Pro23His Transgenic Rats (Line 3) Involves Autophagic and Necroptotic Mechanisms
Source: Front Neurosci. 2020 Nov 3;14:581579. doi: 10.3389/fnins.2020.581579 (PMC7670078; doi:10.3389/fnins.2020.581579)
Supplement: Supplementary Figure 3 — Gating strategy for distinguishing different retinal cell populations, based on size and viability at time of isolation. [file Data_Sheet_3.docx]

Supplementary Material


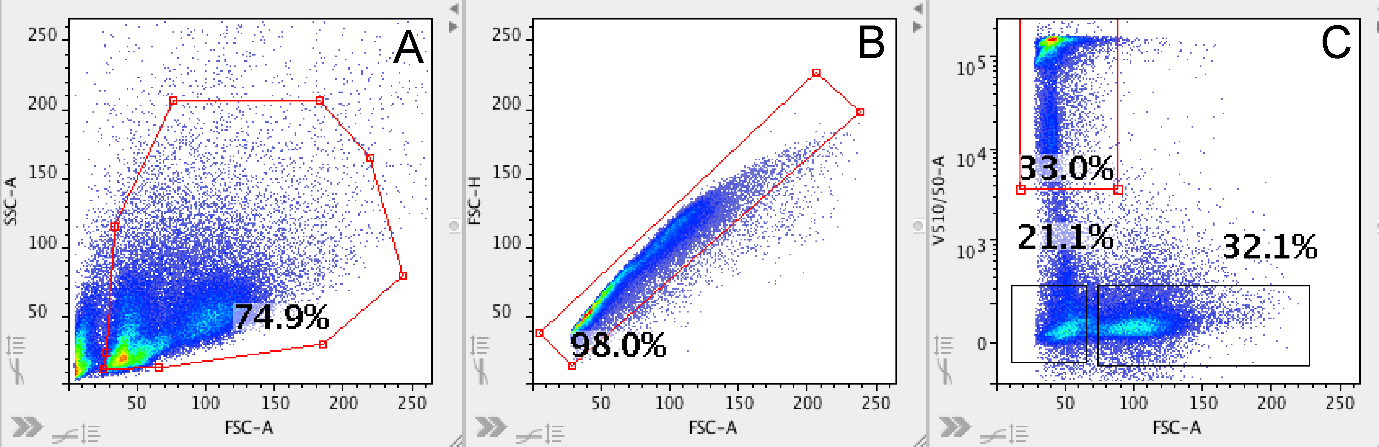


**Supplementary Figure S3. Gating strategy for distinguishing different retinal cell populations, based on size and viability at time of isolation.** For flow cytometry analysis, an initial gate was set on forward scatter (FSC) and side scatter (SSC) to eliminate debris (**A**) and find single cell events for each sample (**B**). Using physical parameters (FSC) and viability staining (DAPI), three distinct subpopulations of cells were consistently detectable in all samples: small dead cells, small live and large live cells (**C**).
