# Supplementary material for: Photoreceptor Degeneration in Pro23His Transgenic Rats (Line 3) Involves Autophagic and Necroptotic Mechanisms
Source: Front Neurosci. 2020 Nov 3;14:581579. doi: 10.3389/fnins.2020.581579 (PMC7670078; doi:10.3389/fnins.2020.581579)
Supplement: Supplementary Figure 4 — Ingenuity pathway modeling of apoptosis gene expression data. [file Data_Sheet_4.docx]

Supplementary Material


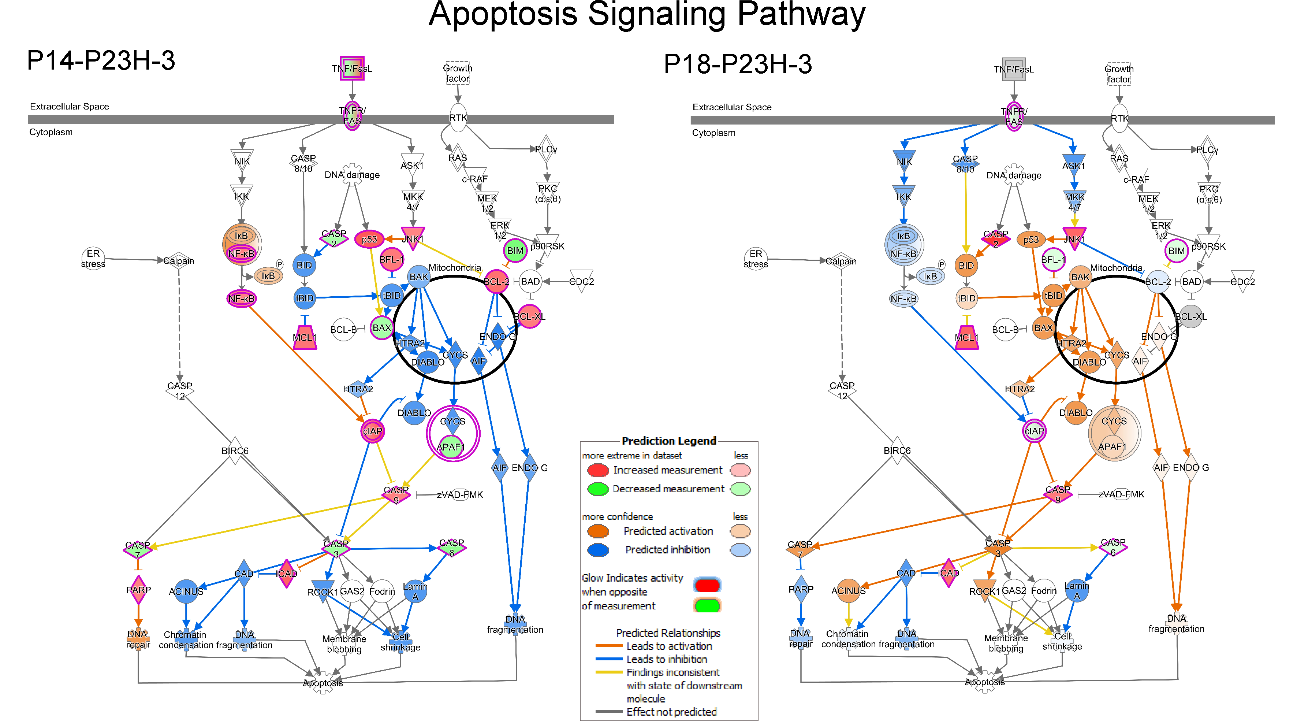


**Supplementary Figure S4. Ingenuity pathway modelling of apoptosis gene expression data.** Ingenuity IPA (Qiagen) analysis of the expression changes from P14 to P18 in genes associated with the canonical apoptotic pathway predicted there were increases in activation of the mitochondrial-associated pathway at P18. there. In this pathway, genes that are upregulated are shown in red and those downregulated are shown in green. The software utilizes the differential expression data and published data on gene/protein interactions to predict pathway components that are activated (orange) or inhibited (blue).
