# Supplementary material for: Photoreceptor Degeneration in Pro23His Transgenic Rats (Line 3) Involves Autophagic and Necroptotic Mechanisms
Source: Front Neurosci. 2020 Nov 3;14:581579. doi: 10.3389/fnins.2020.581579 (PMC7670078; doi:10.3389/fnins.2020.581579)
Supplement: Supplementary Figure 5 — Ingenuity pathway modeling of autophagy gene expression data. [file Data_Sheet_5.docx]

Supplementary Material


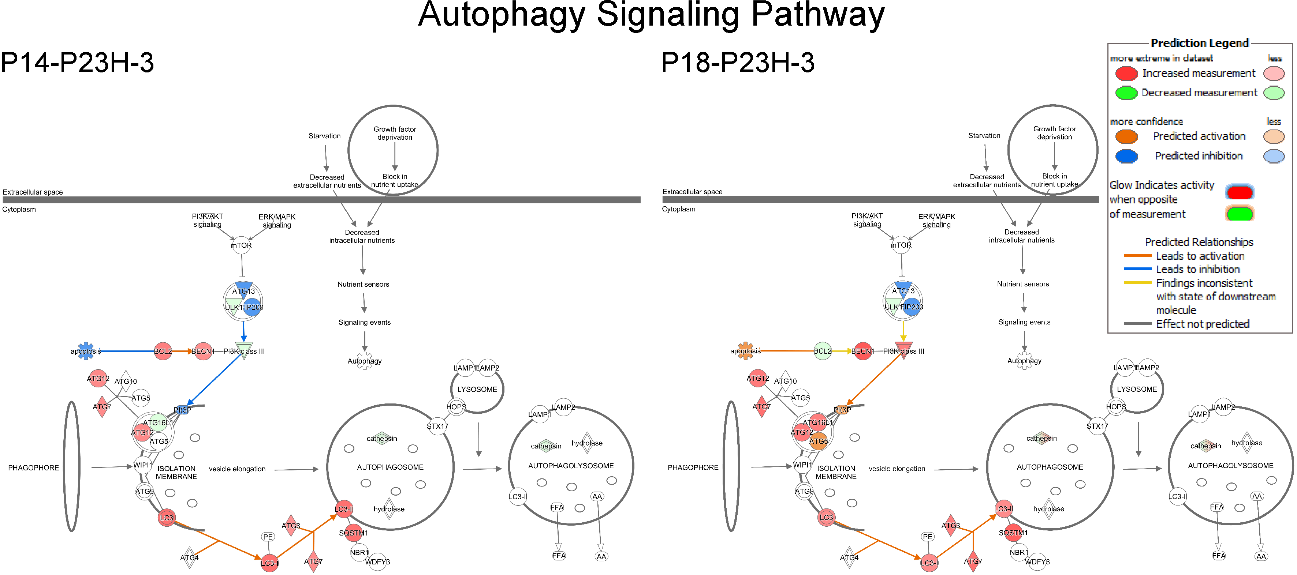


**Supplementary Figure S5. Ingenuity pathway modelling of autophagy gene expression data.** Ingenuity IPA (Qiagen) analysis of the expression changes from P14 to P18 in genes associated with the canonical autophagy pathway predicted activation of this pathway at both P14 and 18. Upregulated genes are shown in red, downregulated genes in green and genes/proteins predicted to be activated (orange) or inhibited (blue).
