# Supplementary material for: Photoreceptor Degeneration in Pro23His Transgenic Rats (Line 3) Involves Autophagic and Necroptotic Mechanisms
Source: Front Neurosci. 2020 Nov 3;14:581579. doi: 10.3389/fnins.2020.581579 (PMC7670078; doi:10.3389/fnins.2020.581579)
Supplement: Supplementary Figure 6 — Ingenuity pathway modeling of necrosis gene expression data. [file Data_Sheet_6.docx]

Supplementary Material


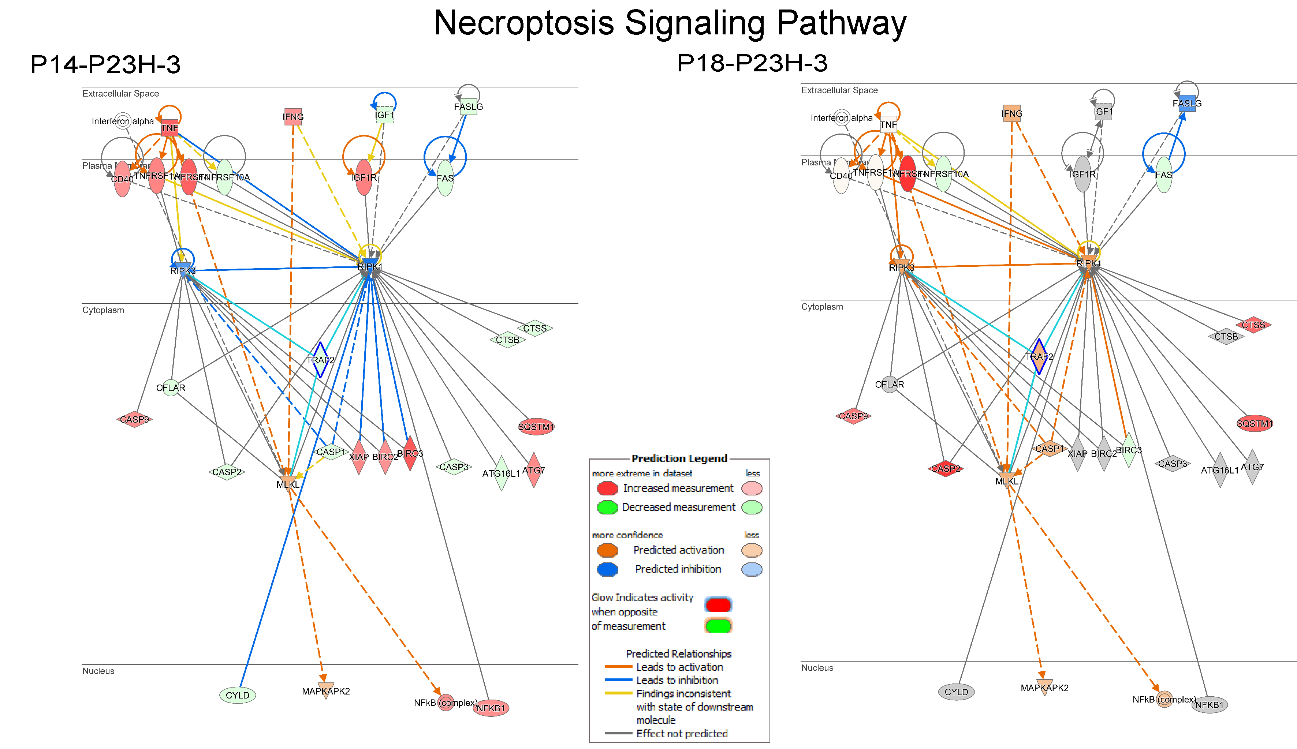


**Supplementary Figure S6. Ingenuity pathway modelling of necrosis gene expression data.** Ingenuity IPA (Qiagen) analysis of the expression changes from P14 to P18 in genes associated with the necroptosis signaling predicted increasing activation of RIPK1 and RIPK3 in this pathway from P14 to P18. Upregulated genes are shown in red, downregulated genes in green and genes/proteins predicted to be activated (orange) or inhibited (blue).
