# Supplementary material for: Photoreceptor Degeneration in Pro23His Transgenic Rats (Line 3) Involves Autophagic and Necroptotic Mechanisms
Source: Front Neurosci. 2020 Nov 3;14:581579. doi: 10.3389/fnins.2020.581579 (PMC7670078; doi:10.3389/fnins.2020.581579)
Supplement: Supplementary Figure 7 — Mitochondrial membrane potential (Δψm) in P23H-3 and SD retinal cells at P14. [file Data_Sheet_7.docx]

Supplementary Material


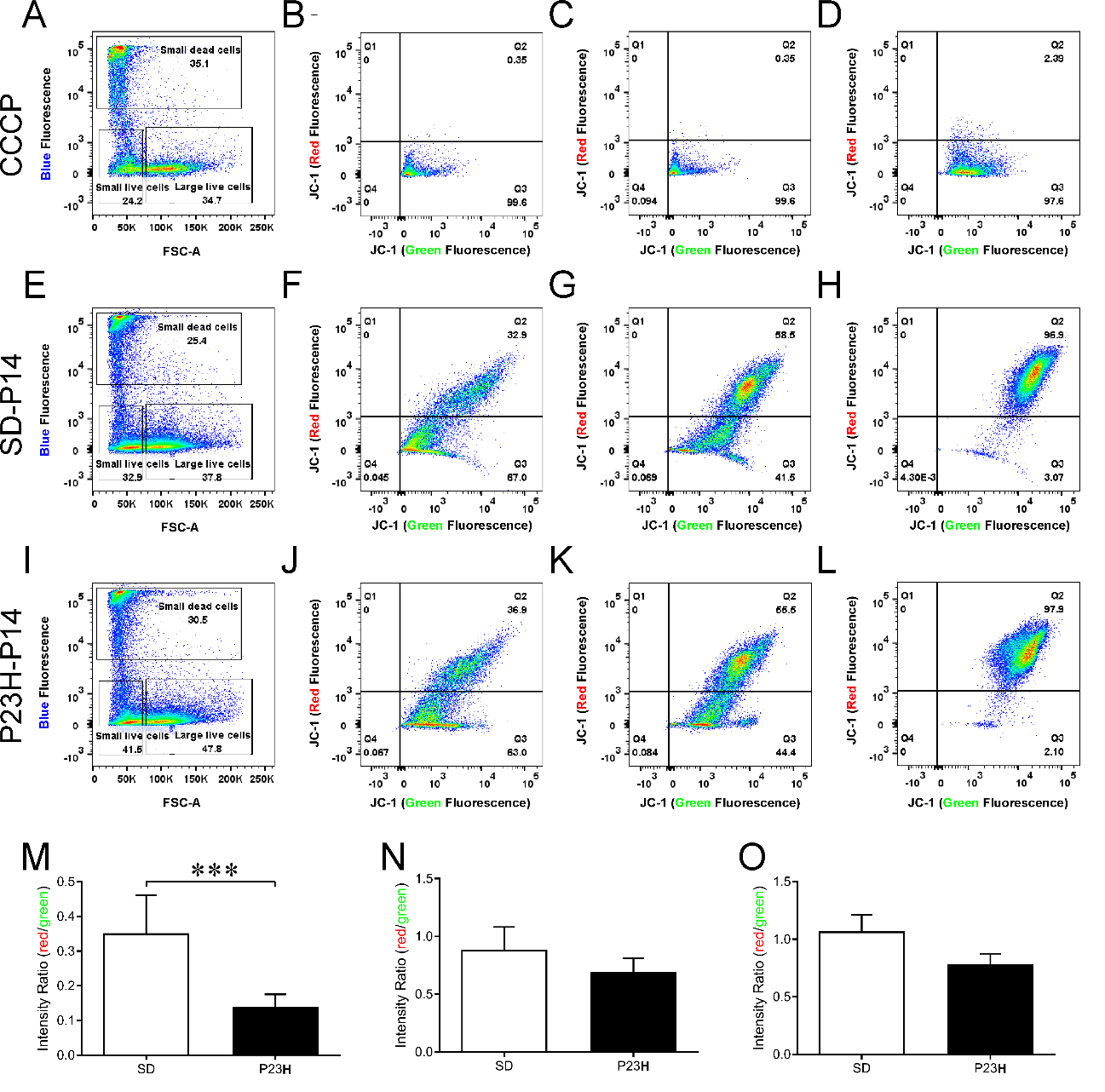


**Supplementary Figure S7. Mitochondrial membrane potential (Δψm) in P23H-3 and SD retinal cells at P14.** Gating strategy for the analysis of retinal cells with different mitochondrial membrane potentials (**A-E** and **I**). Similar ratios of all three distinct subpopulations of cells were detected in all samples: small dead cells (**B, F, J**), small live cells (**C, G, K**) and large live cells (**D, H, L**). Representative plots show levels of polarized (red fluorescence) and depolarized (green fluorescence) mitochondria in CCCP-treated (**B-D**), SD (**F-H**) and P23H-3 (**J-L**) retinal cells. While no major shifts could be detected for small live and large live cells between SD and P23H-3 samples, there appeared to be a slight shift from red- green for the small dead cells (Arrowheads; **F** and **J**). Quantification of red:green fluorescence intensity ratio in the three cell populations from P23H-3 and SD samples showed that small dead (**M**), but not small live (**N**) or large live cells (**O**), had a significant drop in red:green fluorescence intensity ratio (p < 0.0001; 2-tailed unpaired *t*-test) in the P23H-3 retinae. Data presented as mean ± SEM, Student’s t-test, n = 6 in each group.
