# Supplementary material for: Photoreceptor Degeneration in Pro23His Transgenic Rats (Line 3) Involves Autophagic and Necroptotic Mechanisms
Source: Front Neurosci. 2020 Nov 3;14:581579. doi: 10.3389/fnins.2020.581579 (PMC7670078; doi:10.3389/fnins.2020.581579)
Supplement: Supplementary Table 1 — Quantitative PCR analyses of flow cytometry separated cell populations. [file Table_1.docx]

Supplementary Material

**Supplementary Table S1:** Quantitative PCR analyses of flow cytometry separated cell populations. Data shown are the average ΔCt, 2^-ΔCt^ and fold change (FC) for each photoreceptor gene when comparing small dead cell (SDC), small live cell (SLC) and large live cell (LLC) populations. Rhodopsin as a marker for rod photoreceptors was enriched 1.6 to 1.8 fold in the small dead cell populations compared to the other two populations.

| **Gene** | **Av. Normalized ΔCt** | | **2^-ΔC_t_** | | **FC** |
| --- | --- | --- | --- | --- | --- |
|  | **SDC** | **SLC** | **SDC** | **SLC** | **SDC/SLC** |
| ***Rho*** | -6.3 | -5.5 | 79.5 | 43.7 | 1.82 |
| ***Opn1mw*** | 0.7 | 1.2 | 0.6 | 0.4 | 1.40 |
| ***Opn1sw*** | 0.3 | 0.5 | 0.8 | 0.7 | 1.18 |
|  | **SDC** | **LLC** | **SDC** | **LLC** | **SDC/LLC** |
| ***Rho*** | -6.3 | -5.6 | 79.5 | 49.1 | 1.62 |
| ***Opn1mw*** | 0.7 | 0.9 | 0.6 | 0.5 | 1.17 |
| ***Opn1sw*** | 0.3 | 0.1 | 0.8 | 0.9 | 0.90 |
|  | **SLC** | **LLC** | **SLC** | **LLC** | **SLC/LLC** |
| ***Rho*** | -5.5 | -5.6 | 43.7 | 49.1 | 0.89 |
| ***Opn1mw*** | 1.2 | 0.9 | 0.4 | 0.5 | 0.84 |
| ***Opn1sw*** | 0.5 | 0.1 | 0.7 | 0.9 | 0.77 |
