# Supplementary material for: Photoreceptor Degeneration in Pro23His Transgenic Rats (Line 3) Involves Autophagic and Necroptotic Mechanisms
Source: Front Neurosci. 2020 Nov 3;14:581579. doi: 10.3389/fnins.2020.581579 (PMC7670078; doi:10.3389/fnins.2020.581579)
Supplement: Supplementary Table 2 — Antibodies and fixation conditions used in immunofluorescence. [file Table_2.docx]

Supplementary Material

**Supplementary Table S2:** Antibodies used in reverse-phase protein arrays (RPPA).

| **Protein Name** | **Entrez Gene ID (Human)** | **Gene Name** | **Swiss-Prot Acc (Human)** | **Host Species Ig** | **Cross- reactivity** | **Supplier** | **Cat. #** |
| --- | --- | --- | --- | --- | --- | --- | --- |
| Phospho-Src Family (Tyr416) | 6714 | SRC | P12931 | Rabbit IgG | H M R | Cell Signaling Technology | #2101 |
| Src | 6714 | SRC | P12931 | Rabbit IgG | H M R Mk B Pg | Cell Signaling Technology | #2109 |
| Phospho-S6 Ribosomal Protein (Ser235/236) | 6194 | RPS6 | P62753 | Rabbit IgG | H M R Mk | Cell Signaling Technology | #2211 |
| Phospho-S6 Ribosomal Protein (Ser240/244) | 6194 | RPS6 | P62753 | Rabbit IgG | H M R Mk Z | Cell Signaling Technology | #2215 |
| IRS-1 | 3667 | IRS1 | P35568 | Rabbit IgG | H M R | Cell Signaling Technology | #2382 |
| Phospho-IRS-1 (Ser636/639) | 3667 | IRS1 | P35568 | Rabbit IgG | H M R | Cell Signaling Technology | #2388 |
| Bcl-xL | 598 | BCL2L1 | Q07817 | Rabbit IgG | H M R Mk | Cell Signaling Technology | #2762 |
| Phospho-Akt (Thr308) | 207 | AKT1 | P31749;P31751;Q9Y243 | Rabbit IgG | H M R Mk | Cell Signaling Technology | #2965 |
| Phospho0FAK (Tyr397) | 5747 | PTK2 | Q05397 | Rabbit IgG | H M R Pg | Cell Signaling Technology | #3283 |
| FAK | 5747 | PTK2 | Q05397 | Rabbit IgG | H M R Mk B Pg | Cell Signaling Technology | #3285 |
| Phospho-Akt (Ser473) (D9E) XP | 10000 | AKT3 | P31749;P31751;Q9Y243 | Rabbit IgG | H M R Mk B Dm Z | Cell Signaling Technology | #4060 |
| Mcl-1 | 4170 | MCL1 | Q07820 | Rabbit IgG | H M Mk | Cell Signaling Technology | #5453 |
| p70 S6 Kinase | 6198 | RPS6KB1 | P23443 | Rabbit IgG | H M R Mk | Cell Signaling Technology | #9202 |
| Akt | 207 | AKT1 | P31749;P31751;Q9Y243 | Rabbit IgG | H M R Mk Pg C B | Cell Signaling Technology | #9272 |
| Phospho-p53 (Ser15) | 7157 | TP53 | P04637 | Rabbit IgG | H M R Mk | Cell Signaling Technology | #9284 |
| PTEN | 5728 | PTEN | P60484 | Rabbit IgG | H M R Mk | Cell Signaling Technology | #9552 |
| Prohibitin | 5245 | PHB | P35232 | Rabbit IgG | H M R | Santa Cruz | #sc-28259 |
| Bax | 581 | BAX | Q07812 | Rabbit IgG | H M R Mk | Cell Signaling Technology | #2772 |
| Caspase 3 | 836 | CASP3 | P42574 | Rabbit IgG | H M R Mk | Cell Signaling Technology | #9662 |
| Cleaved Caspase-3 (Asp175) (5A1E) | 836 | CASP3 | P42574 | Rabbit IgG | H M R Mk | Cell Signaling Technology | #9664 |
| Cleaved Caspase-7 (Asp198) | 840 | CASP7 | P55210 | Rabbit IgG | H M R Mk | Cell Signaling Technology | #9491 |
| E-Cadherin | 999 | CDH1 | P12830 | Rabbit IgG | H M | Cell Signaling Technology | #3195 |
| IGF-1 Receptor beta | 3480 | IGF1R | P08069 | Rabbit IgG | H M R Mk | Cell Signaling Technology | #3018 |
| IkB alpha | 4792 | NFKBIA | P25963 | Rabbit IgG | H M R Hm Mk Mi | Cell Signaling Technology | #4812 |
| p44/42 MAPK (ERK1/2) | 5595 | MAPK3 | P27361;P28482 | Rabbit IgG | H M R Hm Mk Mi Z B Pg Sc | Cell Signaling Technology | #9102 |
| ROCK1 | 6093 | ROCK1 | Q13464 | Rabbit IgG | H M R Mk | Cell Signaling Technology | #4035 |
| XIAP | 331 | XIAP | P98170 | Rabbit IgG | H Mk | Cell Signaling Technology | #2045 |
| S6 Ribosomal Protein | 6194 | RPS6 | P62753 | Rabbit IgG | H M R Mk | Cell Signaling Technology | #2217 |
| Lck | 3932 | LCK | P06239 | Rabbit IgG | H M | Cell Signaling Technology | #2752 |
| JNK2 | 5599 | MAPK8 | P45983 | Rabbit IgG | H M R Mk | Cell Signaling Technology | #9258 |
| Phospho-p44/42 MAPK (Erk1/2) (Thr202/Tyr204) (D13.14.4E) XP | 5594 | MAPK1 | P27361;P28482 | Rabbit IgG | H M R Mk Pg b Dm Z Dg | Cell Signaling Technology | #4370 |
| Bcl-2 | 596 | BCL2 | P10415 | Rabbit IgG | H M | Cell Signaling Technology | #3498S |
| Rb | 5925 | RB1 | P06400 | Mouse IgG2a | H Mk B Pg | Cell Signaling Technology | #9309 |
| Phospho-Rb (Ser807/811) | 5925 | RB1 | P06400 | Rabbit IgG | H R Mk | Cell Signaling Technology | #9308 |
| Phospho-NF-κB p65 (Ser536) | 5970 | RELA | Q04206 | Rabbit IgG | H M R Mk | Cell Signaling Technology | #3031 |
| Phospho-IκBα (Ser32) (14D4) | 4792 | NFKBIA | P25963 | Rabbit IgG | H M R Mk | Cell Signaling Technology | #2859 |
| SNAIL | 6615 | SNAI1 | O95863 | Rabbit IgG | H M R | Thermofisher | #MA514801 |
| SLUG | 6591 | SNAI2 | O43623 | Rabbit IgG | H M | Thermofisher | #PA186737 |
| TWIST1 | 7291 | TWIST1 | Q15672 | Rabbit IgG | H | Thermofisher | #711565 |
| SMAD1 | 4086 | SMAD1 | Q15797 | Rabbit IgG | H M | Thermofisher | #701168 |
| SMAD2 | 4087 | SMAD2 | Q15796 | Rabbit IgG | H | Thermofisher | #700048 |
| SMAD3 | 4088 | SMAD3 | P84022 | Rabbit IgG | H | Thermofisher | #511500 |
| Fibronectin | 2335 | FN1 | P02751 | Rabbit IgG | H M | Thermofisher | #PA529578 |
| MMP9 | 4318 | MMP9 | P14780 | Rabbit IgG | H M | Thermofisher | #PA513199 |
| CD44 | 960 | CD44 | P16070 | Rabbit IgG | H | Thermofisher | #PA521419 |
| CD24 | 100133941 | CD24 | P25063 | Rabbit IgG | H M R | Thermofisher | #PA549555 |
| Occludin | 100506658 | OCLN | Q16625 | Rabbit IgG | H M | Thermofisher | #710192 |
| Claudin 3 | 1365 | CLDN3 | O15551 | Rabbit IgG | H M R | Thermofisher | #PA516867 |
| Claudin 4 | 1364 | CLDN4 | O14493 | Rabbit IgG | H M | Thermofisher | #PA526621 |
| Claudin 7 | 1366 | CLDN7 | O95471 | Rabbit IgG | H M | Thermofisher | #349100 |
| Cytokeratin 7 | 3855 | KRT7 | P08729 | Rabbit IgG | H | Thermofisher | #PA534764 |
| EpCAM | 4072 | EPCAM | P16422 | Rabbit IgG | H | Thermofisher | #710524 |
| FOXC2 | 2303 | FOXC2 | Q99958 | Mouse IgG1 | H | Thermofisher | #MA5-17077 |
| AMPK alpha-2 | 5563 | PRKAA2 | P54646 | Rabbit IgG | H M R | Thermofisher | #PA5-21494 |
| JNK1/JNK2 /JNK3 | 5601 | MAPK10 | P53779;P45983;P45984 | Rabbit IgG | H M R | Thermofisher | #PA5-38289 |
| FOXM1 | 2305 | FOXM1 | Q08050 | Rabbit IgG | H | Thermofisher | #711695 |
| Bim | 10018 | BCL2L11 | O43521 | Rabbit IgG | H | Thermofisher | #701853 |
| p53 | 7157 | TP53 | P04637 | Mouse IgG1 | H M | Thermofisher | #MA5-11296 |
| phospho NBS1 (Ser343) | 4683 | NBN | O60934 | Rabbit IgG | H | Thermofisher | #PA5-78070 |
| Phospho- FOXO3A (Ser318) | 2309 | FOXO3 | O43524 | Rabbit IgG | H M R | Thermofisher | #PA5-64677 |
| N-cadherin | 1000 | CDH2 | P19022 | Rabbit IgG | H M R Mk Z B | Thermofisher | #PA5-17526 |
| Phospho- Aurora A (Thr288) | 6790 | AURKA | O14965 | Rabbit IgG | H M | Thermofisher | #44-1210G |
