# Supplementary material for: Photoreceptor Degeneration in Pro23His Transgenic Rats (Line 3) Involves Autophagic and Necroptotic Mechanisms
Source: Front Neurosci. 2020 Nov 3;14:581579. doi: 10.3389/fnins.2020.581579 (PMC7670078; doi:10.3389/fnins.2020.581579)
Supplement: Supplementary Table 3 — Antibodies used in reverse-phase protein arrays (RPPA). [file Table_3.docx]

Supplementary Material

**Supplementary Table S3:** Antibodies and fixation conditions used in immunofluorescence experiments

| **Protein Name** | **Entrez Gene ID**  **(Human)** | **Gene Name** | **Swiss-Prot Acc# (Human)** | **Host Species Ig** | **Cross- reactivity** | **Supplier** | **Cat. #** | **Fixation** | **Antigen retrieval** | **Dilution** |
| --- | --- | --- | --- | --- | --- | --- | --- | --- | --- | --- |
| Cleaved Caspase-3 (Asp175) (5A1E) | 836 | *CASP3* | P42574 | Rabbit IgG | H M R Mk | Cell Signaling Technology | #9664 | 4%PFA 30 minutes; frozen sections | 10 mM Na-citrate, pH6; 10 min @95°C | 1:100 |
| MLKL_P Ser345 | 197258 | *MLKL* | Q8NB16 | Rabbit IgG | M | Abcam | ab196436 | 4%PFA 30 minutes; frozen sections | Nil | 1:100 |
| AIF | 9131 | *AIFM1* | O95831 | Mouse IgG | H, R, M, Mk | Sigma-Aldrich | SAB5300151 | Davidson’s fixation; paraffin sections | 10 mM Na-citrate, pH9; 10 min @95°C | 1:100 |
| MTCO1 | 4512 | *MTCO1* | P00395 | Mouse IgG | H, R, M, Mk | Abcam | ab14705 | 4%PFA 30 minutes; frozen sections | Nil | 1:5000 |
| BECN1 | 8678 | *BECN1* | Q14457 | Rabbit IgG | H, R, M | Novus | NB500-249 | 4%PFA 30 minutes; frozen sections | Nil | 1:100 |
| ATG5 | 9474 | *ATG5* | Q9H1Y0 | Rabbit IgG | H, R, M, | Cell Signaling Technology | #12994 | 4%PFA 30 minutes; frozen sections | Nil | 1:100 |
| RIPK3 | 11035 | *RIPK3* | Q9Y572 | Rabbit IgG | H, R, M | Sigma-Aldrich | PRS2283 | 4%PFA 30 minutes; frozen sections | Nil | 1:50 |
