# Supplementary material for: Photoreceptor Degeneration in Pro23His Transgenic Rats (Line 3) Involves Autophagic and Necroptotic Mechanisms
Source: Front Neurosci. 2020 Nov 3;14:581579. doi: 10.3389/fnins.2020.581579 (PMC7670078; doi:10.3389/fnins.2020.581579)
Supplement: Supplementary Table 4 — Quantitative RT-PCR array data for cell death genes expressed in SD and P23H-3 rat retinae at P14. [file Table_4.docx]

Supplementary Material

**Supplementary Table S4:** Quantitative RT-PCR array data for cell death genes expressed in SD and P23H rat retinae at P14.

| **Symbol** | **Average Ct** | | **Average Delta (Ct) (Ct(GOI) - Ave Ct(HKG))** | | **Fold Regulation** | **P value** | **Symbol** | **Average Ct** | | **Average Delta (Ct) (Ct(GOI) - Ave Ct(HKG))** | | **Fold Regulation** | **P value** |
| --- | --- | --- | --- | --- | --- | --- | --- | --- | --- | --- | --- | --- | --- |
|  | **P23H** | **SD** | **P23H** | **SD** |  |  |  | **P23H** | **SD** | **P23H** | **SD** |  |  |
| **Abl1** | 22.97 | 23.24 | 3.902 | 4.124 | 1.167 | 0.311 | **Gadd45a** | 22.07 | 22.28 | 3.003 | 3.164 | 1.119 | 0.559 |
| **Akt1** | 22.08 | 21.84 | 3.009 | 2.718 | -1.224 | 0.242 | **Galnt5** | 33.74 | 33.96 | 14.674 | 14.843 | 1.125 | 0.690 |
| **Apaf1** | 24.04 | 23.86 | 4.967 | 4.742 | -1.169 | 0.068 | **Grb2** | 21.23 | 21.4 | 2.162 | 2.285 | 1.090 | 0.425 |
| **App** | 19.61 | 19.86 | 0.544 | 0.739 | 1.145 | 0.007 | **Hspbap1** | 25.2 | 25.64 | 6.137 | 6.526 | 1.310 | 0.014 |
| **Atg12** | 23.22 | 23.39 | 4.156 | 4.277 | 1.087 | 0.637 | **Htt** | 23.4 | 23.7 | 4.327 | 4.584 | 1.195 | 0.035 |
| **Atg16l1** | 23.48 | 23.47 | 4.412 | 4.356 | -1.040 | 0.832 | **Ifng** | 34.93 | 34.98 | 15.858 | 15.866 | 1.006 | 0.967 |
| **Atg3** | 22.06 | 22.13 | 2.988 | 3.017 | 1.020 | 0.786 | **Igf1** | 22.17 | 22.2 | 3.106 | 3.085 | -1.015 | 0.901 |
| **Atg5** | 23.37 | 23.63 | 4.301 | 4.509 | 1.156 | 0.243 | **Igf1r** | 22.76 | 23.05 | 3.687 | 3.931 | 1.185 | 0.006 |
| **Atg7** | 23.99 | 24.15 | 4.927 | 5.035 | 1.078 | 0.443 | **Ins2** | 32.53 | 31.18 | 13.466 | 12.059 | -2.653 | 0.276 |
| **Atp6v1g2** | 21.62 | 22.02 | 2.550 | 2.904 | 1.279 | 0.030 | **Irgm** | 28 | 28.22 | 8.933 | 9.097 | 1.121 | 0.575 |
| **Bax** | 22.67 | 22.64 | 3.604 | 3.522 | -1.058 | 0.163 | **Kcnip1** | 22.73 | 22.7 | 3.661 | 3.580 | -1.058 | 0.152 |
| **Bcl2** | 26.79 | 27.08 | 7.726 | 7.966 | 1.181 | 0.365 | **Mag** | 29.25 | 29.57 | 10.180 | 10.452 | 1.207 | 0.030 |
| **Bcl2a1** | 29.55 | 29.75 | 10.481 | 10.635 | 1.113 | 0.702 | **Map1lc3a** | 21.29 | 21.75 | 2.225 | 2.637 | 1.330 | 0.129 |
| **Bcl2l1** | 22.33 | 22.5 | 3.266 | 3.382 | 1.084 | 0.382 | **Mapk8** | 22.47 | 22.54 | 3.403 | 3.425 | 1.016 | 0.849 |
| **Bcl2l11** | 30.12 | 29.53 | 11.055 | 10.413 | -1.561 | 0.102 | **Mcl1** | 20.78 | 21.05 | 1.716 | 1.935 | 1.164 | 0.047 |
| **Becn1** | 21.77 | 21.88 | 2.701 | 2.763 | 1.044 | 0.726 | **Nfkb1** | 23.57 | 23.67 | 4.500 | 4.549 | 1.035 | 0.633 |
| **Birc2** | 22.5 | 22.56 | 3.429 | 3.444 | 1.010 | 0.904 | **Nol3** | 24.15 | 24.8 | 5.085 | 5.680 | 1.511 | 0.069 |
| **Birc3** | 31.22 | 31.9 | 12.155 | 12.782 | 1.545 | 0.558 | **Olr1583** | 35 | 34.37 | 15.932 | 15.248 | -1.606 | 0.378 |
| **Bmf** | 23.79 | 24.19 | 4.718 | 5.068 | 1.275 | 0.141 | **Parp1** | 22.79 | 22.89 | 3.722 | 3.772 | 1.036 | 0.640 |
| **Casp1** | 28.68 | 28.5 | 9.616 | 9.387 | -1.172 | 0.490 | **Parp2** | 23.52 | 23.47 | 4.450 | 4.348 | -1.073 | 0.396 |
| **Casp2** | 24.25 | 24.21 | 5.186 | 5.096 | -1.064 | 0.939 | **Pik3c3** | 23.69 | 23.62 | 4.625 | 4.507 | -1.085 | 0.646 |
| **Casp3** | 22.36 | 22.2 | 3.289 | 3.083 | -1.153 | 0.044 | **Pten** | 20.63 | 20.61 | 1.562 | 1.491 | -1.050 | 0.453 |
| **Casp6** | 25.77 | 25.41 | 6.706 | 6.291 | -1.332 | 0.057 | **Pvr** | 27.02 | 27.4 | 7.951 | 8.280 | 1.256 | 0.066 |
| **Casp7** | 26.33 | 26.15 | 7.257 | 7.034 | -1.167 | 0.867 | **Rab25** | 31.22 | 30.62 | 12.155 | 11.506 | -1.568 | 0.429 |
| **Casp9** | 24.45 | 24.57 | 5.383 | 5.451 | 1.049 | 0.328 | **RGD1311517** | 26.13 | 26.03 | 7.058 | 6.914 | -1.105 | 0.500 |
| **Cd40** | 28.73 | 28.78 | 9.664 | 9.665 | 1.001 | 0.986 | **Rps6kb1** | 22.93 | 22.56 | 3.860 | 3.439 | -1.338 | 0.369 |
| **Cd40lg** | 35 | 35 | 15.932 | 15.882 | -1.035 | 0.586 | **Snca** | 20.72 | 20.72 | 1.653 | 1.600 | -1.037 | 0.328 |
| **Cflar** | 24.43 | 24.45 | 5.366 | 5.334 | -1.023 | 0.650 | **Spata2** | 22.75 | 23.26 | 3.682 | 4.142 | 1.376 | 0.026 |
| **Commd4** | 22.3 | 22.57 | 3.230 | 3.455 | 1.170 | 0.146 | **Sqstm1** | 21.76 | 22.23 | 2.693 | 3.113 | 1.339 | 0.004 |
| **Ctsb** | 20.26 | 20.16 | 1.196 | 1.040 | -1.114 | 0.127 | **Sycp2** | 30.76 | 30.59 | 11.690 | 11.471 | -1.164 | 0.496 |
| **Ctss** | 23.44 | 23.49 | 4.376 | 4.369 | -1.004 | 0.934 | **Tmem57** | 27.15 | 27.38 | 8.082 | 8.262 | 1.133 | 0.532 |
| **Cybb** | 28.75 | 28.8 | 9.678 | 9.685 | 1.005 | 0.954 | **Tnf** | 33.01 | 33.61 | 13.938 | 14.489 | 1.465 | 0.316 |
| **Cyld** | 27.21 | 27.23 | 8.142 | 8.116 | -1.019 | 0.901 | **Tnfrsf10b** | 28.84 | 28.65 | 9.776 | 9.536 | -1.181 | 0.285 |
| **Defb1** | 31.77 | 32.01 | 12.704 | 12.887 | 1.136 | 0.519 | **Tnfrsf11b** | 27.51 | 28.33 | 8.437 | 9.210 | 1.708 | 0.017 |
| **Dennd4a** | 21.79 | 21.79 | 2.726 | 2.671 | -1.038 | 0.458 | **Tnfrsf1a** | 26.19 | 26.42 | 7.121 | 7.307 | 1.138 | 0.624 |
| **Dffa** | 24.4 | 24.84 | 5.329 | 5.718 | 1.310 | 0.051 | **Tnfrsf4** | 29.22 | 29.82 | 10.155 | 10.702 | 1.462 | 0.123 |
| **Dpysl4** | 20.87 | 21.47 | 1.800 | 2.348 | 1.463 | 0.010 | **Tnfrsf8** | 31.27 | 31.69 | 12.204 | 12.574 | 1.292 | 0.674 |
| **Esr1** | 30.25 | 30.7 | 11.181 | 11.582 | 1.321 | 0.159 | **Tp53** | 23.56 | 24.08 | 4.495 | 4.965 | 1.385 | 0.022 |
| **Fas** | 30.11 | 30.03 | 11.039 | 10.909 | -1.094 | 0.679 | **Traf2** | 23.59 | 23.48 | 4.527 | 4.364 | -1.120 | 0.283 |
| **Faslg** | 28.91 | 28.95 | 9.837 | 9.830 | -1.005 | 0.796 | **Txnl4b** | 23.36 | 23.37 | 4.289 | 4.255 | -1.023 | 0.792 |
| **Foxi1** | 35 | 34.12 | 15.932 | 15.006 | -1.899 | 0.243 | **Ulk1** | 23.69 | 23.59 | 4.618 | 4.476 | -1.104 | 0.334 |
| **Gaa** | 22.95 | 22.91 | 3.887 | 3.792 | -1.067 | 0.881 | **Xiap** | 19.96 | 20.15 | 0.894 | 1.036 | 1.104 | 0.349 |
