# Supplementary material for: Photoreceptor Degeneration in Pro23His Transgenic Rats (Line 3) Involves Autophagic and Necroptotic Mechanisms
Source: Front Neurosci. 2020 Nov 3;14:581579. doi: 10.3389/fnins.2020.581579 (PMC7670078; doi:10.3389/fnins.2020.581579)
Supplement: Supplementary Table 5 — Quantitative RT-PCR array data for cell death genes expressed SD and P23H-3 rat retinae at P18. [file Table_5.docx]

Supplementary Material

**Supplementary Table S5:** Quantitative RT-PCR array data for cell death genes expressed SD and P23H rat retinae at P18.

| **Symbol** | **Average Ct** | | **Average Delta(Ct) (Ct(GOI) - Ave Ct(HKG))** | | **Fold Regulation** | **P value** | **Symbol** | **Average Ct** | | | **Average Delta(Ct) (Ct(GOI) - Ave Ct(HKG))** | | **Fold Regulation** | **P value** |
| --- | --- | --- | --- | --- | --- | --- | --- | --- | --- | --- | --- | --- | --- | --- |
|  | **P23H** | **SD** | **P23H** | **SD** |  |  |  | **P23H** | **SD** | **P23H** | | **SD** |  |  |
| **Abl1** | 22.78 | 23.46 | 3.628 | 4.134 | 1.420 | 0.046 | **Gadd45a** | 21.75 | 22.48 | 2.605 | | 3.156 | 1.465 | 0.008 |
| **Akt1** | 22.11 | 22.27 | 2.965 | 2.941 | -1.017 | 0.790 | **Galnt5** | 34.77 | 32.79 | 15.627 | | 13.458 | -4.498 | 0.033 |
| **Apaf1** | 24.23 | 24.39 | 5.086 | 5.063 | -1.016 | 0.841 | **Grb2** | 21.51 | 21.74 | 2.367 | | 2.409 | 1.030 | 0.759 |
| **App** | 19.85 | 19.75 | 0.704 | 0.425 | -1.214 | 0.247 | **Hspbap1** | 24.98 | 25.76 | 5.836 | | 6.430 | 1.509 | 0.018 |
| **Atg12** | 23.12 | 23.6 | 3.970 | 4.268 | 1.229 | 0.192 | **Htt** | 23.27 | 23.89 | 4.123 | | 4.559 | 1.353 | 0.216 |
| **Atg16l1** | 23.05 | 23.47 | 3.899 | 4.142 | 1.184 | 0.034 | **Ifng** | 35 | 35 | 15.853 | | 15.672 | -1.134 | 0.530 |
| **Atg3** | 21.88 | 22.33 | 2.730 | 3.000 | 1.206 | 0.014 | **Igf1** | 22.46 | 22.53 | 3.313 | | 3.203 | -1.080 | 0.265 |
| **Atg5** | 23.46 | 23.87 | 4.313 | 4.543 | 1.172 | 0.041 | **Igf1r** | 22.51 | 22.64 | 3.361 | | 3.310 | -1.036 | 0.618 |
| **Atg7** | 24.07 | 24.65 | 4.920 | 5.319 | 1.319 | 0.224 | **Ins2** | 31.83 | 31.65 | 12.686 | | 12.320 | -1.288 | 0.511 |
| **Atp6v1g2** | 21.5 | 21.92 | 2.354 | 2.592 | 1.180 | 0.069 | **Irgm** | 28.04 | 28.16 | 8.894 | | 8.832 | -1.044 | 0.771 |
| **Bax** | 22.98 | 23.08 | 3.830 | 3.755 | -1.054 | 0.690 | **Kcnip1** | 22.88 | 23.22 | 3.734 | | 3.892 | 1.116 | 0.225 |
| **Bcl2** | 26.77 | 26.86 | 7.620 | 7.535 | -1.061 | 0.747 | **Mag** | 28.87 | 29.97 | 9.722 | | 10.638 | 1.886 | 0.160 |
| **Bcl2a1** | 30.78 | 30.17 | 11.638 | 10.844 | -1.734 | 0.035 | **Map1lc3a** | 22.16 | 22.43 | 3.011 | | 3.102 | 1.065 | 0.745 |
| **Bcl2l1** | 22.28 | 22.97 | 3.131 | 3.641 | 1.425 | 0.127 | **Mapk8** | 22.2 | 22.89 | 3.051 | | 3.559 | 1.422 | 0.009 |
| **Bcl2l11** | 30.2 | 29.72 | 11.051 | 10.391 | -1.581 | 0.020 | **Mcl1** | 20.76 | 21.47 | 1.614 | | 2.145 | 1.445 | 0.013 |
| **Becn1** | 21.61 | 22.41 | 2.464 | 3.083 | 1.536 | 0.038 | **Nfkb1** | 23.62 | 23.97 | 4.473 | | 4.644 | 1.125 | 0.453 |
| **Birc2** | 22.46 | 22.7 | 3.311 | 3.376 | 1.046 | 0.902 | **Nol3** | 24.31 | 24.92 | 5.163 | | 5.591 | 1.346 | 0.052 |
| **Birc3** | 31.8 | 30.33 | 12.656 | 11.006 | -3.140 | 0.043 | **Olr1583** | 35 | 34.11 | 15.853 | | 14.784 | -2.098 | 0.082 |
| **Bmf** | 22.94 | 23.58 | 3.796 | 4.247 | 1.367 | 0.343 | **Parp1** | 23.18 | 23.18 | 4.037 | | 3.853 | -1.136 | 0.573 |
| **Casp1** | 28.96 | 28.82 | 9.818 | 9.489 | -1.256 | 0.095 | **Parp2** | 23.37 | 23.66 | 4.225 | | 4.333 | 1.078 | 0.543 |
| **Casp2** | 23.8 | 24.88 | 4.649 | 5.556 | 1.875 | 0.011 | **Pik3c3** | 23.3 | 23.73 | 4.153 | | 4.403 | 1.189 | 0.082 |
| **Casp3** | 22.45 | 22.88 | 3.303 | 3.552 | 1.188 | 0.061 | **Pten** | 20.27 | 20.72 | 1.121 | | 1.390 | 1.205 | 0.058 |
| **Casp6** | 26.31 | 26.17 | 7.163 | 6.839 | -1.252 | 0.025 | **Pvr** | 27.06 | 27.25 | 7.909 | | 7.919 | 1.006 | 0.907 |
| **Casp7** | 26.49 | 26.56 | 7.340 | 7.230 | -1.079 | 0.881 | **Rab25** | 30.74 | 30.82 | 11.591 | | 11.497 | -1.068 | 0.588 |
| **Casp9** | 24.43 | 24.92 | 5.283 | 5.592 | 1.238 | 0.027 | **RGD1311517** | 26.34 | 26.28 | 7.196 | | 6.953 | -1.183 | 0.412 |
| **Cd40** | 29.42 | 29.5 | 10.276 | 10.171 | -1.075 | 0.836 | **Rps6kb1** | 22.49 | 23.04 | 3.346 | | 3.714 | 1.291 | 0.321 |
| **Cd40lg** | 34.2 | 35 | 15.055 | 15.672 | 1.533 | 0.335 | **Snca** | 21.1 | 21.29 | 1.955 | | 1.961 | 1.004 | 0.985 |
| **Cflar** | 24.43 | 24.67 | 5.283 | 5.344 | 1.043 | 0.647 | **Spata2** | 23.03 | 23.81 | 3.884 | | 4.485 | 1.517 | 0.126 |
| **Commd4** | 21.93 | 22.53 | 2.783 | 3.206 | 1.340 | 0.164 | **Sqstm1** | 21.8 | 22.54 | 2.657 | | 3.216 | 1.473 | 0.014 |
| **Ctsb** | 20.14 | 20.23 | 0.998 | 0.898 | -1.072 | 0.191 | **Sycp2** | 29.79 | 30.22 | 10.645 | | 10.892 | 1.186 | 0.545 |
| **Ctss** | 22.98 | 23.64 | 3.835 | 4.314 | 1.393 | 0.007 | **Tmem57** | 26.91 | 27.56 | 7.767 | | 8.231 | 1.379 | 0.117 |
| **Cybb** | 28.51 | 28.46 | 9.366 | 9.132 | -1.177 | 0.298 | **Tnf** | 32.83 | 32.74 | 13.680 | | 13.415 | -1.202 | 0.880 |
| **Cyld** | 26.78 | 27.11 | 7.635 | 7.783 | 1.108 | 0.132 | **Tnfrsf10b** | 29.61 | 28.25 | 10.459 | | 8.921 | -2.905 | 0.006 |
| **Defb1** | 31.97 | 27.83 | 12.819 | 8.497 | -20.008 | 0.001 | **Tnfrsf11b** | 28.44 | 28.28 | 9.290 | | 8.951 | -1.265 | 0.376 |
| **Dennd4a** | 21.57 | 21.76 | 2.420 | 2.435 | 1.011 | 0.955 | **Tnfrsf1a** | 26.66 | 26.24 | 7.509 | | 6.910 | -1.514 | 0.186 |
| **Dffa** | 24.83 | 25.45 | 5.685 | 6.124 | 1.356 | 0.013 | **Tnfrsf4** | 28.78 | 29.89 | 9.635 | | 10.561 | 1.900 | 0.046 |
| **Dpysl4** | 21.15 | 21.89 | 2.008 | 2.563 | 1.469 | 0.047 | **Tnfrsf8** | 31.01 | 31.69 | 11.863 | | 12.360 | 1.411 | 0.236 |
| **Esr1** | 30.29 | 29.61 | 11.143 | 10.285 | -1.813 | 0.148 | **Tp53** | 24.65 | 24.38 | 5.501 | | 5.055 | -1.363 | 0.053 |
| **Fas** | 29.68 | 28.5 | 10.530 | 9.176 | -2.557 | 0.006 | **Traf2** | 23.4 | 23.8 | 4.250 | | 4.468 | 1.163 | 0.190 |
| **Faslg** | 28.59 | 28.76 | 9.438 | 9.436 | -1.002 | 0.887 | **Txnl4b** | 23.43 | 24.19 | 4.287 | | 4.857 | 1.484 | 0.148 |
| **Foxi1** | 35 | 35 | 15.853 | 15.672 | -1.134 | 0.530 | **Ulk1** | 23.63 | 23.76 | 4.487 | | 4.430 | -1.040 | 0.616 |
| **Gaa** | 22.4 | 22.52 | 3.249 | 3.187 | -1.044 | 0.762 | **Xiap** | 19.9 | 20.25 | 0.752 | | 0.918 | 1.122 | 0.037 |

References - Supplementary Materials

1. Fulton, A.B.; Hansen, R.M.; Findl, O. The development of the rod photoresponse from dark-adapted rats. *Investigative ophthalmology & visual science* **1995**, *36*, 1038-1045.

2. Machida, S.; Kondo, M.; Jamison, J.A.; Khan, N.W.; Kononen, L.T.; Sugawara, T.; Bush, R.A.; Sieving, P.A. P23h rhodopsin transgenic rat: Correlation of retinal function with histopathology. *Investigative ophthalmology & visual science* **2000**, *41*, 3200-3209.
